# Application of TiO_2_ in Photocatalytic Bacterial Inactivation: Review

**DOI:** 10.3390/ijms262110593

**Published:** 2025-10-30

**Authors:** Vesna Lazić, Valentina Nikšić, Jovan M. Nedeljković

**Affiliations:** Vinča Institute of Nuclear Sciences—National Institute of the Republic of Serbia, Centre of Excellence for Photoconversion, University of Belgrade, 11001 Belgrade, Serbia; vesna.lazic@vin.bg.ac.rs (V.L.); valentina.niksic@vin.bg.ac.rs (V.N.)

**Keywords:** TiO_2_, photocatalytic antibacterial activity, reactive oxygen species, TiO_2_-based structures, optical properties, surface properties

## Abstract

Photocatalytic pathogen inactivation is gaining increasing importance due to the rising number of microbial species resistant to conventional antibacterial agents. Titanium dioxide (TiO_2_)-based photocatalysts have emerged as a promising solution, being not only potent antibacterial agents but also environmentally friendly and capable of simultaneously degrading organic pollutants. This review summarizes recent advances in the antibacterial performance of different TiO_2_ modifications, including commercial nanopowders, nanoparticles with various morphologies, thin films, composites, and polymer-supported nanostructures, all primarily activated under UV light. Given the limited ability of pristine TiO_2_ to harvest solar radiation, we also highlight the most recent strategies for designing visible-light-responsive TiO_2_, such as doping, incorporation of plasmonic metal nanoparticles, formation of heterostructures, and interfacial charge transfer complexes. In addition, we discuss the fundamental structural features of TiO_2_, the mechanisms of reactive oxygen species (ROS) generation involved in bacterial inactivation, and kinetic models describing antibacterial efficiency. These insights aim to advance the understanding and development of eco-friendly, cost-effective, and sustainable photocatalytic disinfection technologies.

## 1. Introduction

**Figure 1:**
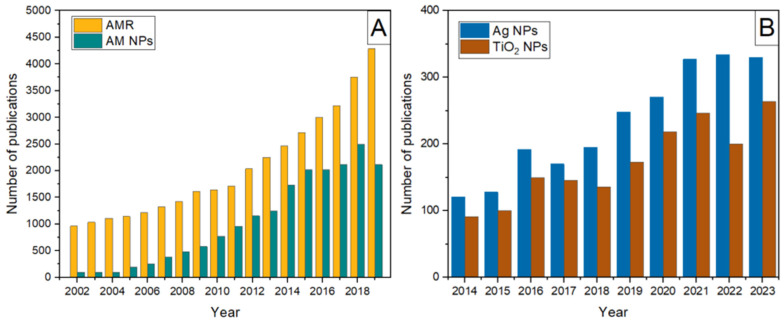
(**A**) The annual growth of publications on antimicrobial resistance in the environment and antimicrobial nanoparticles over the long-term period. The figure was generated by the authors based on data extracted and combined from references [2] (Figure 1) and [4] (Figure 1). (**B**) The number of publications in 2014–2023 focused on silver nanoparticles as antimicrobial agents and TiO_2_ applications in medicine and as antimicrobial agents. The figure was generated by the authors using data from references [8] (Figure 2A) and [27] (Figure 1A).

Antimicrobial resistance (AMR) is a commonly used term that describes a phenomenon in which microbial organisms (bacteria, fungi, and viruses) become less susceptible to the antimicrobial medicines used to eliminate or control them [1]. Over the years, increased AMR of microbial species to conventional antibacterial reagents, reflected by the rising number of reports on infections non-responsive to first-line antimicrobials [2] (Figure 1A, curve a), has urged the search for alternative strategies that avoid resistance mechanisms, leading to the Political Declaration on AMR by the United Nations General Assembly in 2016.

Still in development, nanotechnology has created novel antibacterial systems to replace organic antibiotics, whether synthetic or natural extracts, which decompose fast and sometimes require high dosages, leaving pollutant residues [3]. Bibliometric data [4] indicated a significant increase in publications on antimicrobial nanoparticles during the second decade of this century, but with several years of lag time compared to recognition of the problem (compare curves a and b in Figure 1A).

Since ancient times, silver and silver compounds have been recognized as potent biocides and antibacterial agents. However, because air and light cause uncontrollable reduction processes, soluble silver compounds are not appropriate for use. Silver nanoparticles with the desired shape and surface property, suitable for effective use as an antibacterial agent, can now be synthesized due to recent advancements in sophisticated synthetic processes [5,6,7]. Figure 1B shows the publication rate concerning the use of silver nanoparticles as antimicrobial agents from 2014 to 2023 [8]. From the technological standpoint, the use of immobilized silver nanoparticles instead of free-standing ones is preferred; the silver nanoparticles have been either attached to or embedded in various inorganic and organic supports, such as zeolite [9], clay [10], hydroxyapatite [11], paper [12], and various polymers, including textile fibers [13,14,15,16,17,18,19,20,21,22]. The mechanism behind the antibacterial activity of silver particles is not entirely understood because of the co-occurrence of silver ions and solid particulates. However, according to recent studies, the toxicity of silver particles is mostly, if not entirely, caused by dissolved silver ions [23,24,25]. Because silver particles serve as a reservoir for silver ions, colloquially, this mechanism is known as the “*Trojan horse*”. Generally, the antibacterial activity of silver is strongly size-dependent (the smaller the particles, the higher the antibacterial efficiency), concentration-dependent, and exposure time-dependent. However, due to the growing use of silver particles in many items, along with their mass production and inappropriate disposal, the accumulation of silver poses a risk to the environment and, consequently, to human health [26].

As evidenced by the growing number of research articles in recent years (Figure 1B), titanium dioxide (TiO_2_) is the most widely used photocatalyst for inactivating microbial species because of its low cost, physicochemical properties (stability, durability, and corrosion resistance), biocompatibility, reusability, the ability to control its morphology (size and shape) and interface, and the high reactivity of photogenerated electron-hole pairs [27]. The antimicrobial activity of TiO_2_ relies on charge carriers that reach and get trapped at the TiO_2_ surface. Photogenerated electrons and holes react with electron acceptors and donors, respectively, forming highly reactive oxygen species (ROS), such as superoxide radical anion ($O_{2}^{⦁-}$), hydroxyl radicals (HO^•^), hydrogen peroxide (H_2_O_2_), and singlet oxygen (^1^O_2_). Detailed information concerning the formation and properties of ROS is provided in the following section. It should not be forgotten that ROS can oxidize organic molecules, which has recently led to an enormous effort by many research groups to develop TiO_2_-based photocatalysts capable of simultaneously photocatalytic degradation of organic pollutants and inactivation of microbial species [28,29,30,31].

**Figure 2:**
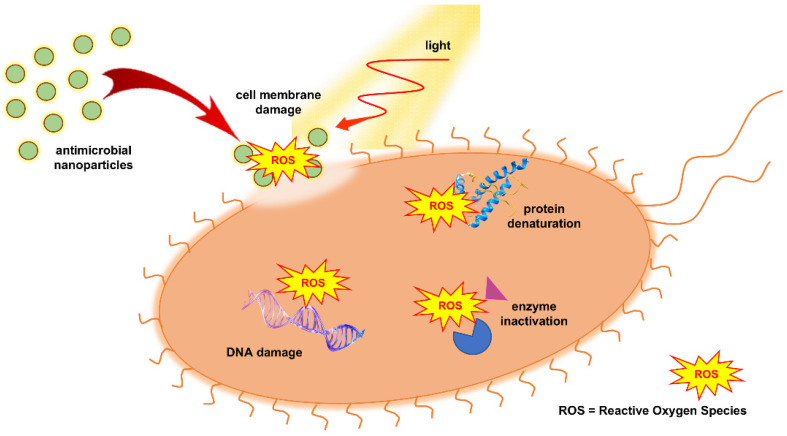
Schematic representation of the photocatalytic inactivation mechanism of bacterial cells by TiO_2_.

Figure 2 illustrates the photocatalytic inactivation mechanism of bacterial species. Bacterial inactivation primarily occurs through irreversible damage to the cell wall and membrane caused by reactive oxygen species (ROS) generated on the TiO_2_ surface. TiO_2_ nanoparticles are mainly localized on the outer bacterial membrane, where direct contact facilitates oxidative attack and membrane disruption. Only after the structural integrity of the cell wall is compromised can ROS, and occasionally a small fraction of nanoparticles, penetrate the cytoplasm, leading to the leakage of intracellular components, cell lysis, and eventual mineralization of the cell [32]. Efficient antimicrobial action, therefore, requires close contact between TiO_2_ particles and pathogens. As shown by van Loosdrecht et al. [33], bacterial surfaces typically possess a negative charge under physiological conditions, making electrostatic attraction a dominant factor in nanoparticle adhesion.

In addition to oxidative damage to cell membranes and proteins, TiO_2_ photocatalysis also induces direct oxidative injury to nucleic acids. Both chromosomal and plasmid DNA are vulnerable to hydroxyl radical (^•^OH) and superoxide anion (O_2_^•−^) attack, resulting in single- and double-strand breaks, base oxidation, and cross-linking reactions that prevent replication and transcription [34,35,36,37]. Studies have shown that supercoiled plasmid DNA exposed to UV-irradiated TiO_2_ transforms first into the relaxed and then linear form, confirming strand scission [34,35]. Recent work further demonstrated that ROS generated on TiO_2_ surfaces can degrade extracellular and plasmid-borne antibiotic resistance genes (ARGs), thereby suppressing horizontal gene transfer between bacteria [37]. This dual effect, microbial inactivation and genetic material degradation, highlights the potential of TiO_2_-based photocatalysis not only for disinfection but also for reducing the environmental spread of antibiotic resistance.

The cell wall structure has a strong influence on the interaction between TiO_2_ nanoparticles and bacteria. Gram-positive bacteria, characterized by a thick peptidoglycan layer and absence of an outer membrane, generally show lower susceptibility to photocatalytic inactivation due to limited TiO_2_ adhesion and ROS penetration. In contrast, Gram-negative bacteria possess a thinner peptidoglycan layer and an outer membrane rich in lipopolysaccharides, which promotes electrostatic attraction of TiO_2_ particles and facilitates oxidative damage. These structural differences are often reflected in higher inactivation rates observed for Gram-negative species such as *E. coli* compared to Gram-positive bacteria like *S. aureus* [38].

Another factor influencing bacterial susceptibility to photocatalytic inactivation is the activity of superoxide dismutase (SOD), an enzyme responsible for converting superoxide radicals (O_2_^•−^) into less reactive hydrogen peroxide [39]. The level of SOD expression varies among bacterial species, with generally higher activity observed in Gram-positive bacteria, contributing to their greater resistance to oxidative stress. Moreover, exposure to TiO_2_ nanoparticles may alter SOD expression, either by enzyme inhibition due to excessive ROS or by temporary upregulation as part of the bacterial stress response.

This review summarizes and discusses the mechanism and antibacterial action of different TiO_2_ forms of materials, starting with commercial up to sophisticated TiO_2_-based architectures. Since the main obstacle for efficient pathogen inactivation is TiO_2_’s low harvesting ability of solar radiation, we paid special attention to various strategies to achieve a visible-light-responsive TiO_2_ photocatalyst, including doping, heterostructures with small bandgap semiconductors, and the formation of interfacial charge transfer complexes. Consequently, this review article aims to provide a critical assessment of the current state-of-the-art research on the antibacterial properties of TiO_2_-based materials, with a primary focus on their potential benefits for preserving the ecosystem.

## 2. TiO_2_—Basic Properties

Among transition metal oxides, TiO_2_ is one of the most studied materials. TiO_2_ has a wide range of industrial applications in the paint, plastic, food, cosmetics, hygiene, and paper industries, as well as agriculture, gas sensing, medical devices, and medication delivery. A great deal of scientific and engineering interest in photoinduced catalytic processes induced the groundbreaking work done by Fujishima and Honda [40] in the early 1970s on the water splitting reaction in a photoelectrochemical system using a TiO_2_ photoanode under UV light excitation. Besides the water splitting reaction [41,42,43], another energy-related application of TiO_2_ is dye-sensitized solar cells, which were discovered by O’Regan and Grätzel [44]. The second type of TiO_2_ photocatalytic use is environmental remediation, including both the photocatalytic destruction of organic molecules [45,46,47,48] and the inactivation of microbial species [27,49] due to the TiO_2_’s strong photocatalytic oxidation capability. Over the years, numerous review articles have offered a thorough examination of TiO_2_ properties. Therefore, we only discussed the properties of TiO_2_ that are associated with its antibacterial action, directing readers to additional information in the following review articles [50,51,52].

### 2.1. Structure

**Figure 3:**
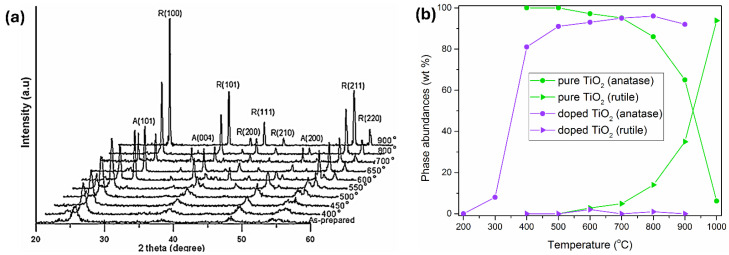
(**a**) XRD patterns of the as-prepared sample and heat-treated; the peaks labeled as A and R represent the anatase and rutile phases, respectively [48], (**b**) Phase abundances of anatase and rutile as a function of temperature for pure and Cr-doped TiO_2_ nanotubes [49].

It is well-known that TiO_2_ has four naturally occurring polymorphs: anatase and rutile with a tetragonal structure, brookite with an orthorhombic structure, and uncommon TiO_2_(B) with a monoclinic structure [53]. In this review, anatase and rutile crystal structures are the only ones under consideration. Since anatase is a low-density, metastable phase, it easily transforms into the rutile phase at high temperatures [54,55]. The XRD plots of pristine TiO_2_ before and after thermal annealing in air at different temperatures are shown in Figure 3a. Figure 3a reveals that the main crystalline structure below 550 °C is anatase. Then, at 550 °C, an emerging rutile phase emerges, whose content slowly increases up to 100% at 800 °C without any trace of the anatase phase. So, in the temperature range between 550 and 750 °C, the heat treatments generate mixtures of anatase and rutile phases. In addition, the rutile phase has sharp XRD peaks, while the sharpening of anatase peaks with the increase in heating temperature is a consequence of an increase in the crystallite size.

Besides inducing phase transformation, heat treatment affects morphology, crystallinity, porosity, and specific surface area of TiO_2_ and leads to the loss of surface hydroxyl groups and the removal of organic materials at high temperatures (≥400 °C). So, vice versa, anatase-rutile transformation is not only temperature dependent but also depends on many factors, such as particle size and shape, the presence of dopants or impurities, and their concentrations. An instructive example is a study by Low et al. [55] concerning the crystallization behavior of pure and Cr-doped TiO_2_ nanotubes prepared by the anodization process. The phase abundances of anatase and rutile as a function of temperature in pure TiO_2_ nanotube arrays are shown in Figure 3b. So, the anatase phase appears at 400 °C from amorphous TiO_2_ nanotubes, and its abundance decreases with an increase in temperature due to the appearance of the rutile phase at 600 °C. After 800 °C, the abundance of the rutile phase rapidly increases at the expense of the anatase phase. However, although its abundance is low, the anatase phase remains present even at 1000 °C. On the other hand, in the presence of Cr dopant, the rapid formation of the anatase phase occurs when the annealing temperature rises from 300 to 400 °C and continues to increase until 900 °C (see Figure 3b). However, the abundance of the rutile phase in the high-temperature range is negligible, suggesting that, without getting into an explanation, the rutile phase is unstable.

### 2.2. Surface Charge

One of the prerequisites for efficient photocatalytic reactions is the adsorption of reactants on the semiconductor surface. It is well-known that TiO_2_ and other metal oxides suspended in water are amphoteric, so surface hydroxyl groups undergo the following acid–base equilibria:

(1) $$Ti_{sur}-OH_{2}^{+}\overset{pK_{a1}}{\Leftrightarrow }Ti_{sur}-OH\overset{pK_{a2}}{\Leftrightarrow }Ti_{sur}-O^{-}$$

The pH of the zero point of charge (pH_ZPC_) is one-half of the pK_a1_ and pK_a2_:

(2) $$pH_{ZPC}=\frac{pK_{a1}+pK_{a2}}{2}$$

Based on the extensive literature survey by Kosmulski [56], the average and median values of pH_ZPC_ for anatase are 5.9 and 6, respectively, while for rutile, they are 5.4 and 5.5, respectively. Knowing that bacteria have a negative surface charge [33], their adhesion to the TiO_2_ surface at pH < pH_ZPC_ is favored.

Although the subject of this review is the photocatalytic inactivation of pathogens by TiO_2_, several reports indicated the inactivation of microbial species by TiO_2_ in the dark [57,58,59,60]. For example, Pagnout et al. [57] reported that the cell viability of *E. coli* is severely affected at pH 5.5 due to their accumulation onto the TiO_2_ surface, facilitated by the electrostatic interaction between positively charged TiO_2_ surface hydroxyl groups and negatively charged bacterial walls. This phenomenon was observed at a much lower degree at pH 7.0, close to the pH_ZPC_ of TiO_2_, while at pH 9.5, it is negligible since both TiO_2_ and cells have negatively charged surfaces.

### 2.3. Energy Level Alignment—Optical Properties

Anatase has a large bandgap of 3.2 eV, while the bandgap of rutile is slightly smaller, at 3.0 eV. So, TiO_2_ absorbs less than 5% of the available solar light photons, allowing only UV photons to produce electron-hole pairs and stimulate redox processes on the catalyst surface. Because of that, there has been tremendous interest in recent years in improving the optical response of TiO_2_ and other metal oxides by bringing their absorption to a more practical visible spectral range, which we will discuss in detail. In addition to the limitless use of solar energy, the advantage of photocatalysis is the possibility of carrying on photo-driven heterogeneous reactions under mild experimental conditions (room temperature and atmospheric pressure).

**Figure 4:**
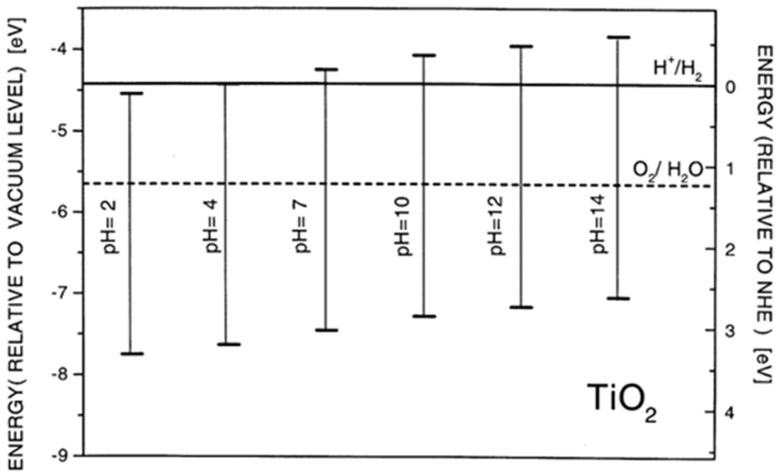
The pH-dependent energy level position of VB_max_ and CB_min_ in anatase toward the vacuum level and the normal hydrogen electrode [61].

Besides the ability to harvest solar light, the energy level alignment is an equally important semiconductor characteristic that determines its photocatalytic ability. The pH-dependent position of the valence band maximum (VB_max_) and the conduction band minimum (CB_min_) in anatase, related to H^+^/H_2_ and O_2_/H_2_O potentials, is presented in Figure 4 [55]. The VB_max_ and CB_min_ potentials change following the Nernst equation, i.e., −0.059 V per pH unit. Considering the position of VB_max_ and CB_min_ in TiO_2_, the photogenerated holes are powerful oxidants, while photogenerated electrons are good reductants.

## 3. Reactive Oxygen Species (ROS)

The photocatalytic processes involve redox reactions on heterogeneous solid surfaces induced by photogenerated charge carriers. The electron transfer reactions of photogenerated electrons and holes with oxygen and water (hydroxyl ions) are crucial because photocatalytic processes occur in an aqueous environment under aerobic conditions. Thus, oxygen converts into highly reactive species, generally referred to as ROS, which further participate in oxidative and reductive processes. The main ROS are superoxide radical anion ($O_{2}^{•-}$), hydrogen peroxide (H_2_O_2_), singlet oxygen (^1^O_2_), and hydroxyl radical (HO^•^) [62].

### 3.1. Generation of ROS

Excitation of TiO_2_ with photons whose energy exceeds the bandgap energy promotes electrons from the VB to CB, leaving holes behind.

(3) $$TiO_{2}\overset{h\upsilon }{\to }e_{CB}^{-}+h_{VB}^{+}$$

The generation of electron-hole pairs takes place on the femtosecond time scale. Hoffmann et al. [45] proposed a multi-step mechanism for photocatalytic reactions over TiO_2_ based on the laser flash photolysis experiments. Following the generation of charge carriers, in the picosecond to nanosecond time scale, a competition between the trapping and recombination processes of the photogenerated electron-hole pairs occurs. Photogenerated charge carriers can either recombine radiatively, producing light, or non-radiatively, dissipating the absorbed energy as heat. Another competition occurs on a microsecond to millisecond time scale and involves trapped charge carriers, i.e., their recombination versus electron transfer reactions with adsorbed reactants on the TiO_2_ surface. Of course, the effective suppression of parasitic recombination processes is essential to the overall outcome of the photocatalytic process.

The excitation of aerated aqueous dispersions of TiO_2_ leads to ROS formation. Oxygen serves as the primary electron acceptor and transforms into the superoxide radical anion:

(4) $$e_{CB}^{-}+O_{2}\to O_{2}^{•-}$$

The chemical structure of the superoxide radical anion alters with pH due to acid–base equilibrium:

(5) $$O_{2}^{•-}+H^{+}\overset{pK_{a}=4.8}{\Leftrightarrow }HO_{2}^{•}$$

On the other hand, the formation of hydroxyl radicals (HO^•^) takes place in the reaction of holes with adsorbed water or hydroxyl ions:

(6a) $$h_{VB}^{+}+H_{2}O\to HO^{•}+H^{+}$$

(6b) $$h_{VB}^{+}+OH^{-}\to HO^{•}$$

The singlet oxygen (^1^O_2_), whose energy is higher than that of the triplet oxygen with two unpaired electrons, may be generated by the oxidation of superoxide anion radical:

(7) O2•−→−e−O21

However, the ^1^O_2_ formation generally occurs by the energy transfer from the excited state of photosensitizers to the ground state of O_2_ [62]. Since the ^1^O_2_ role is insignificant in the inactivation of pathogens compared to other reactive intermediate species ($O_{2}^{•-}$, H_2_O_2_, and HO^•^), the ^1^O_2_ will not be analyzed further.

The H_2_O_2_ can be formed by the disproportionation of two protonated superoxide radical anions:

(8) $$HO_{2}^{•}+HO_{2}^{•}\to O_{2}+H_{2}O_{2}$$

or by dimerization of hydroxyl radicals:

(9) $$HO^{•}+HO^{•}\to H_{2}O_{2}$$

**Figure 5:**
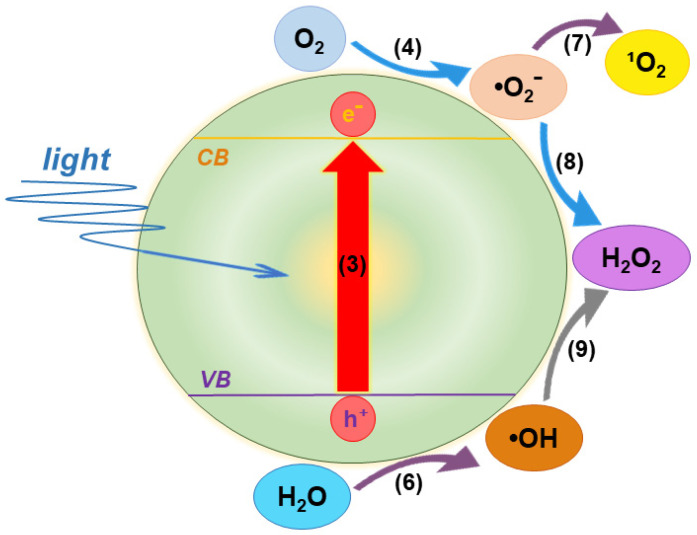
ROS formation upon the excitation of aerated aqueous dispersions of TiO_2_. Numbers in the scheme correspond to the equations presented in the text.

For clarity, the concurrent photocatalytic reduction and oxidation reactions of oxygen and water, leading to the sequential generation of ROS, are illustrated in Figure 5.

### 3.2. Redox Properties of ROS

Detailed information about the redox potentials of free radicals in aqueous solution is collected by Wardman [63] and Hayyan et al. [64]. We provide the redox potentials of ROS at pH 7, which is considered the standard state in biochemistry.

In the literature, the widely accepted redox potential of the O_2_/$O_{2}^{•-}$ couple is −0.33 V *versus* NHE [65]. Since the superoxide radical anion becomes protonated at acid pH (pK_a_ = 4.8), the redox potential of the O_2_/$O_{2}^{•-}$ couple is pH-independent for pH > 4.8. It is worth mentioning that the term “*superoxide*” prompted several scientists to emphasize the high reactivity of $O_{2}^{•-}$.

The further reduction of oxygen, i.e., the reduction in superoxide radical anion to H_2_O_2_, is presented by the following equation:

(10) $$O_{2}^{•-}+2H^{+}+e^{-}\to H_{2}O_{2}$$

At pH 7, the redox potential of the $O_{2}^{•-},2H^{+}/H_{2}O_{2}$ couple is 0.91 V *versus* NHE [66]. Taking into account protonation constants for $O_{2}^{•-}$ and H_2_O_2_ (4.8 and 11.7, respectively), the redox potential slope is twice as steep in the pH range from 4.8 to 11.7 as it is at pH < 4.8 and pH > 11.7, where it changes by −0.059 V per pH unit. H_2_O_2_ has a long shelf life and, compared to superoxide radical anion and hydroxyl radical, is non-reactive when stored in the dark.

On the other hand, the redox potential of the HO^•^/H_2_O couple is +2.72 V *versus* NHE at pH 7 [67]. Thus, the hydroxyl radical is one of the most potent oxidizing species, which reacts fast at a diffusion-controlled rate (~10^10^ M^−1^ s^−1^) [68], producing other radical species through hydroxyl addition and hydrogen abstraction. Buxton et al. [68] have compiled the rate constants for the reactions of hydroxyl radicals (HO^•^ and O^•−^), hydrated electrons, and hydrogen atoms in aqueous solution, as determined by the pulse radiolysis technique.

Since these ROS are the main oxidative agents responsible for disrupting bacterial cell membranes, proteins, and nucleic acids, their generation and reactivity directly determine the antibacterial performance of TiO_2_-based photocatalysts [69].

## 4. Antimicrobial Activity of TiO_2_

Pathogenic microorganisms are widely distributed in environmental matrices such as water, air, soil, and food-related surfaces [2,27]. Their persistence poses serious risks to public health, including outbreaks of gastrointestinal diseases (caused by bacteria such as *Escherichia coli* and *Salmonella*), respiratory infections (e.g., *Legionella pneumophila* in water systems), and hospital-acquired infections linked to contaminated medical surfaces. In addition, fungal contamination in food and air can lead to allergic reactions and mycotoxin-related illnesses. The increasing prevalence of antibiotic-resistant strains in these environments further exacerbates the problem, underscoring the urgent need for novel and sustainable antimicrobial technologies. In this context, TiO_2_-based photocatalysts provide a promising approach for reducing microbial contamination in diverse environmental settings.

Since the majority of pathogenic microorganisms can impair human health by ingestion, inhalation, or skin contact, biological pollution is as essential as non-biological toxins (such as pesticides, antibiotics, and heavy metals). Thus, one of the most critical issues that humanity needs to solve is the efficient and affordable purification of water. Since conventional sterilizing techniques, such as UV radiation, ozonation, and chlorination, all have drawbacks, heterogeneous photocatalysis is the most promising among the various water treatment techniques [2,27].

The development of photocatalytic antibacterial materials has advanced rapidly in the past few decades, as evidenced by several review articles covering various periods and aspects of this field [27,70,71]. This is in response to the groundbreaking work of Matsunaga et al. [72], which showed for the first time that photocatalytic technology can eliminate pathogens, including yeast (*S. cerevisiae*), Gram-positive bacteria (*L. acidophilus*), Gram-negative bacteria (*E. coli)*, and green algae (*C. vulgaris*).

This review covers the antimicrobial performance of various TiO_2_-based materials, starting with commercial and followed by synthesized TiO_2_ with increasing complexity levels. It is well-known that TiO_2_ with desired morphology and phase composition, supported or incorporated into matrices or forming hybrids with inorganic or organic compounds, can be prepared using physical, chemical, and biological methods. Considering the enormous number of research papers concerning the antimicrobial activity of TiO_2_, we constrain this review to the bottom-up approach for TiO_2_ synthesis based on chemical and biological methods. Physical preparation techniques, such as physical milling, physical vapor deposition, sputtering, and laser ablation, are excluded except when the creation of nanostructures in the bottom-up approach relies on physical processes, like deposition.

The antibacterial kinetics of TiO_2_ nanoparticles depend strongly on their physicochemical state and the irradiation conditions. Under dark conditions, bare TiO_2_ exhibits limited antibacterial activity, which is often attributed to surface adsorption of bacterial cells and direct interactions with the cell membrane, leading to localized disruption of membrane integrity. In the case of doped or modified TiO_2_, enhanced dark antibacterial effects have been reported, most likely due to changes in surface charge (zeta potential), increased ion release, or the presence of surface defects that facilitate bacterial adhesion [64].

Under light irradiation, photocatalytic activation of TiO_2_ generates ROS, including hydroxyl radicals (^•^OH), superoxide anion radicals (O_2_^•−^), and hydrogen peroxide (H_2_O_2_). These species induce oxidative stress that damages cell membranes, proteins, and nucleic acids, ultimately leading to bacterial death [3,4,5]. Modified TiO_2_ systems, such as doped materials, plasmonic composites, or heterostructures, exhibit enhanced kinetics due to improved charge separation and extended light absorption into the visible range [63,64,65]. Consequently, the antibacterial efficiency of TiO_2_-based systems arises from the interplay between dark interactions (adsorption and surface effects) and light-driven photocatalytic ROS generation.

### 4.1. Methods for In Vitro Determination of Antibacterial Activity

For photocatalytic TiO_2_ systems, conventional antibacterial assays require modification because TiO_2_ is a solid, light-responsive material. Therefore, test conditions such as catalyst dispersion, irradiation wavelength and dose, and control experiments (dark and photolysis) are essential for accurate interpretation of antibacterial efficiency. Several in vitro methods have been developed to evaluate the antibacterial activity of materials; however, only a few of them are suitable for testing solid photocatalytic systems, such as TiO_2_ [73]. In this section, we briefly describe the experimental approaches most commonly applied for assessing the antibacterial activity of TiO_2_-based photocatalysts, including the modified disk diffusion, dilution, and time-kill methods. These methods are discussed with attention to the specific challenges associated with photocatalytic materials, such as light irradiation parameters, catalyst dispersion, and the need for dark and photolysis controls [74].

#### 4.1.1. Disk-Diffusion Method

The disk-diffusion method is a well-known procedure based on the principle that the antibacterial agent, placed on agar previously inoculated with the test bacterium, diffuses radially outward into the agar and inhibits germination and growth of the test microorganism. The diameters of inhibition growth zones provide qualitative information on the antibacterial activity of the tested compound, which enables the categorization of bacteria as susceptible, intermediate, or resistant [75].

However, the disk-diffusion method is not suitable for determining the minimum inhibitory concentration (MIC), since it provides only qualitative or semi-quantitative information about antibacterial activity and does not allow precise quantification of the effective concentration.

The disk diffusion method is often used as an initial screening tool to assess whether TiO_2_ coatings or nanoparticles inhibit bacterial growth zones. However, due to the limited diffusion of TiO_2_ particles in agar, this method provides only qualitative insights and cannot be used to compare different TiO_2_ modifications reliably.

Nevertheless, the disk-diffusion method has several advantages over other methods, including low cost, the ability to test large numbers of microorganisms and antimicrobial agents, and the straightforward interpretation of the obtained results.

#### 4.1.2. Dilution Tests

The antibacterial activity of antibacterial agents can be determined in vitro using two different types of dilution laboratory tests (agar and broth), which are quantified by the minimum inhibitory concentration (MIC) value. The minimum inhibitory concentration (MIC) of an antibacterial agent is the lowest concentration that prevents the growth of a particular strain of pathogen. The determination of the minimum bactericidal concentration (MBC) of antibacterial agents often follows the determination of the MIC values. The main difference between MBC and MIC is that MBC is the lowest concentration that causes the death of bacterial species. At the same time, the MIC is the lowest concentration of an antibacterial agent that prevents bacterial growth. The ratio between MBC and MIC provides information on whether the antibacterial agent is bactericidal. The antibacterial agent is bactericidal if the MBC and MIC values are close (ratio ≤ 4 times). However, suppose the MBC of the antibacterial agent against the microorganism is ≥ 32 times larger compared to the MIC value. In that case, it is considered that this bacterium is resistant to the tested antibacterial agent.

MIC and MBC assays allow for quantification of bacterial susceptibility to TiO_2_ suspensions or composites. These tests are beneficial when comparing the efficiency of doped vs. undoped TiO_2_ nanoparticles under identical conditions.

The gold standard for determining the MIC, which is often given in μg/mL or mg/mL, is the agar dilution method [76]. With this technique, a series of agar plates with progressively higher concentrations of the target antibacterial drug is prepared, often in doubling dilutions (1, 2, 4, 8, 16, 32 μg/mL, etc.). Each series of plates containing increasing concentrations of the antibacterial agent (the ultimate inoculum is approximately 5 × 10^4^ CFU/spot; CFU stands for colony-forming units) is covered with the suspension of tested microorganisms, which is then incubated for the entire night. The MIC, or the lowest concentration of an antibacterial agent that prevents the development of a particular bacterium, is then determined by analyzing agar plates. The principle of the agar dilution method is the same as the disk-diffusion method. However, it is laborious due to the time required to prepare each set of agar plates for each antibacterial agent of interest. However, for labs that regularly test bacterial isolates against a small number of antibacterial drugs, the agar dilution approach is cost-effective.

The broth dilution method is more versatile and less laborious than the agar dilution method. The primary difference between these two methods lies in the medium in which the antibacterial agent is diluted (agar or broth). Otherwise, the principle of MIC determination is the same. In broth dilution, bacterial growth is assessed visually (turbidity) after 18–24 h of incubation: the first tube or well without visible turbidity is considered the MIC. The broth microdilution version of the broth dilution method can be automated, enabling simultaneous testing of various bacteria with diverse concentrations.

**Figure 6:**
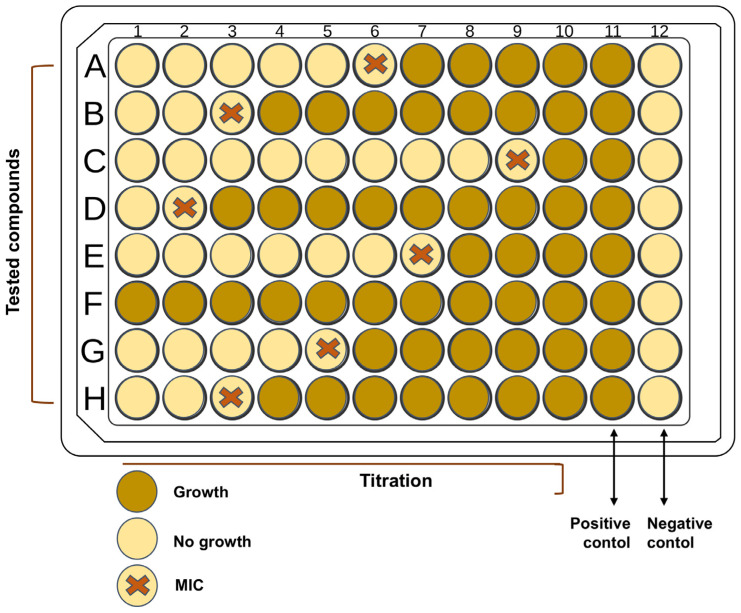
Schematic presentation of the principle for MIC determination by both dilution methods (agar dilution method and broth dilution method).

For clarity, in Figure 6, we schematically presented the principle for MIC determination by both dilution methods (agar dilution method and broth dilution method) for several antibacterial agents (from A to H) with increasing concentrations (from 1 to 10) [77]. Positive control tests are experiments performed without antibacterial agents, while, on the other hand, bacteria are absent in negative control tests. The cross labels the MICs.

#### 4.1.3. Time-Kill Method

The time-kill kinetics assay is a broth-based method that quantifies the killing rate of a known inoculum of microorganisms by an antimicrobial agent, assessed by collecting sequential samples to count survivors. Besides the time-dependent effect of antimicrobial agents on strains of microorganisms, the assay can determine the concentration-dependent effect of antimicrobial agents and synergy or antagonism between two or more antimicrobials.

The percentages of bacterial reduction (R, %) after specific contact times can be calculated using the following equation:

(11) $$R=\frac{C_{0}-C}{C_{0}}\times 100$$

where C_0_ (CFU) and C (CFU) are the number of bacterial colonies in the sample without (control) and in the presence of an antibacterial agent, respectively. Another way to express time-kill curves is to use a log scale for the surviving bacteria in the semi-log diagrams. Knowing that the inactivation of bacterial species by TiO_2_ is facilitated by photogenerated ROS, the two modified positive control tests should be conducted. The first control test, without antibacterial agents, has to be performed under the same illumination conditions to verify that UV light does not directly kill microorganisms. The second control test has to be performed in the dark to estimate the extent of the inactivation of bacteria due to the contact with the TiO_2_ surface, as reported in a few research articles [57,58,59,60].

Time-kill assays provide dynamic information on bacterial inactivation kinetics under photocatalytic irradiation. Such data are critical for evaluating whether TiO_2_ generates sufficient ROS to achieve complete eradication within a clinically relevant timeframe.

Despite their widespread use, there is still no standardized methodology for assessing the antibacterial activity of TiO_2_ materials. Differences in irradiation conditions, bacterial strains, and assay protocols often hinder direct comparison across studies. This highlights the need for harmonized testing strategies to enable meaningful evaluation of TiO_2_-based antimicrobial systems.

However, most studies are limited to cell survival data and rarely include complementary analyses that could clarify the mechanism of inactivation. Parameters such as the level of oxidative stress, membrane integrity, changes in cell morphology, and metabolic activity are still largely unexplored for TiO_2_-based photocatalytic systems.

### 4.2. TiO_2_ Suspensions

Pioneering work by Matsunaga et al. [72] initiated enormous research efforts to study the antimicrobial activity of commercial TiO_2_ (Degussa P25), followed by studies of synthesized TiO_2_ nanoparticles as a function of their properties, mainly phase composition and morphology. Besides Degussa P25, we discuss the antibacterial activity of TiO_2_ particles prepared using chemical synthetic approaches, like colloidal, sol–gel, and hydrothermal, but, on the other hand, we omitted studies with TiO_2_ fabricated by chemical vapor deposition and spray pyrolysis. Also, there are constraints in covering literature concerning the most recent, eco-friendly biological synthetic approach. We restricted this review to TiO_2_ prepared by plant extracts, omitting the use of microbial species.

#### 4.2.1. Antibacterial Activity of Commercial TiO_2_ (Degussa P25)

**Figure 7:**
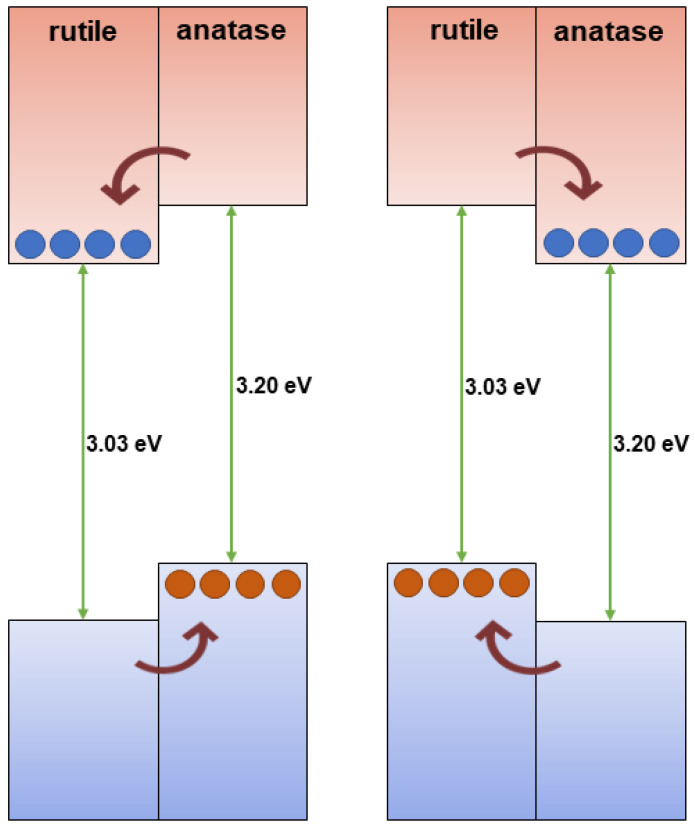
Two proposed valence and conduction band alignments for the anatase/rutile interface.

The most extensively studied photocatalyst is Degussa P25, whose properties, such as phase composition (anatase to rutile ratio is 80 to 20 percent), specific surface area (50 m^2^/g), and primary particle size (~20 nm), are well-documented in the literature [78]. The energy band alignment at the anatase/rutile interface is responsible for the high activity of TiO_2_ P-25 powder in various photocatalytic processes. As seen in Figure 7 (left), there has been a consensus since 1996 that the conduction band of anatase is displaced negatively by 0.2 eV in comparison to the conduction band of rutile, based on electrochemical experiments by Kavan et al. [79]. The transfer of photogenerated electrons from anatase to rutile and the transfer of holes from rutile to anatase are both favored by this energy band alignment. Nonetheless, anatase and rutile have similar valence band locations. However, as illustrated in Figure 7 (right), photogenerated electrons move from rutile into anatase, as demonstrated by photoemission [80] and electron paramagnetic resonance experiments [81] conducted on mixed rutile/anatase samples about ten years later. Numerous density functional theory calculations corroborate these experimental results, showing that the valence band of rutile has an energy that is approximately 0.4 eV higher than that of anatase [82]. In contrast to pure phases, anatase/rutile composites, of which the Degussa P25 is the most widely used benchmark material, display synergistic effects in photoinduced catalytic processes, despite disagreements regarding energy alignment in mixed-phase TiO_2_. Since both heterostructures are type II (staggered gap), the most straightforward explanation for this synergy depends on the band alignment, regardless of which of the two shown in Figure 7 is correct. This increases the efficiency of the separation of photogenerated charge carriers and, in turn, influences their lifetime.

Table 1 summarizes the research articles concerning the antimicrobial activity of Degussa P25 particles suspended in water contaminated by various pathogens. We can conclude the following from a statistical point of view. First, the published articles, references from 83 to 111, cover two decades, the last from the 20th century and the first from the 21st century. To be clear, it does not mean that the scientific community lost interest in Degussa P25; on the contrary, Degussa P25 became the standard used for comparison and evaluation of the antimicrobial ability of newly developed material, since the same experimental conditions of photocatalytic experiments (geometry of photocatalytic reactor, light sources, etc.) in different laboratories are pretty unlikely. Second, *Escherichia coli* (*E. coli*), a Gram-negative bacterium, was the most used pathogen in these studies.

However, we should be aware that *E. coli* is much more sensitive to any disinfecting method than other fecal bacteria, as pointed out by Agulló-Barceló et al. [112], and is not the best choice to indicate microbiological contamination in research studies.

**Figure 8:**
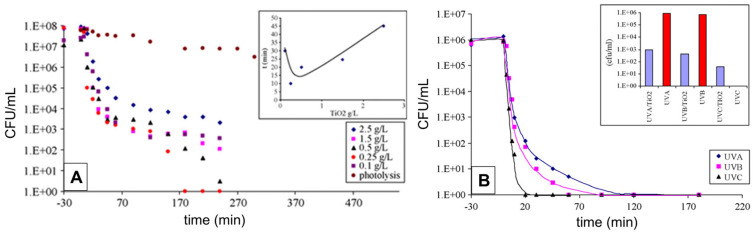
(**A**) Effect of TiO_2_ concentration (Degussa P25) on the inactivation of 10^7^–10^8^ CFU/mL *E. coli* suspensions. Inset: Irradiation time required to decrease the bacteria concentrations from 10^8^ to 10^5^ CFU/mL as a function of TiO_2_ concentration. (**B**) Photocatalytic *E. coli* inactivation in the presence of 0.25 g/L TiO_2_ under UVA, UVB, and UVC illumination. Inset: The concentration of *E. coli* after 10 min of illumination in the presence or absence of TiO_2_ using different UV light [103].

Finding the optimal concentration of Degussa P25 and the optimal light irradiation intensity, as well as the impact of the various UV light spectral domains (UVA (315–400 nm), UVB (280–315 nm), and UVC (100–280 nm)), pH, and temperature, was the main goal of the early research. The work by Benabbou et al. [103] on TiO_2_ Degussa P25’s inactivation of *E. coli* under various physicochemical conditions is informative in this regard, among many others. Figure 8A and Figure 8B, respectively, display typical findings regarding the dependence of *E. coli* inactivation rates on illumination utilizing UV light sources with varying spectral profiles and Degussa P25 concentration. As illustrated in Figure 8B, we stress the significance of comparing microbial inactivation tests for simply photolytic versus photocatalytic experiments. In contrast to the photocatalytic tests using Degussa P25 (inset to Figure 8B), nonsignificant inactivation of *E. coli* was noted in photolytic experiments employing UVA or UVB. However, the UVC photolysis was significantly more effective than the Degussa P25-assisted photocatalytic inactivation of *E. coli*.

#### 4.2.2. Antibacterial Activity of TiO_2_ Prepared by Chemical Methods

The sol–gel process is a versatile solution method for synthesizing ceramic materials at the nanoscale. An in-depth description of the sol–gel process can be found in the review article by Hench et al. [113]. Briefly, the sol–gel process involves the conversion of a sol (colloid) into a solid gel phase (interconnected, rigid nanoparticle network). In the case of TiO_2_, hydrolysis of titanium salts (for example, TiCl_4_) or metal–organic compounds (metal alkoxides (Ti(OR)_4_) leads to the formation of a sol, as shown by Equation (12a) and Equation (12b), respectively:

(12a) $$TiCl_{4}+2H_{2}O\to TiO_{2}+4HCl$$

(12b) $$Ti{\left(OR\right)}_{4}+2H_{2}O\to TiO_{2}+4ROH$$

Then, the TiO_2_ colloid converts into a gel by prolonged storage or drying, and finally, the heat treatment induces the formation of the dense, uniform, ultra-fine powder. Of course, if the concentration of TiO_2_ is low, the gel does not form. After the pH adjustment by dialysis or ultrafiltration, the formation of a stable colloid consisting of nanometer-sized anatase particles takes place.

According to Yoshimura et al. [114], hydrothermal processing is any chemical reaction, whether homogeneous or heterogeneous, that takes place in a closed system at a temperature higher than room temperature and a pressure higher than 1 atm when a solvent, either aqueous or non-aqueous, is present.

The principle of the conventional hydrothermal method and its variants emerged by hybridizing the hydrothermal method with other processes (microwaves, electrochemistry, ultrasound, and mechanochemistry), which broadens the possibility for new materials fabrication and is provided in a review article by Shandilya et al. [115].

The advantage of both methods, sol–gel and hydrothermal, lies in the possibility of conducting tailor-made synthesis of high-purity ceramic materials with desired morphology (shape and size, i.e., specific surface area) and the possibility of depositing them onto different kinds of support or preparing composites. In this section, we will correlate the antibacterial ability of TiO_2_ prepared by the sol–gel or hydrothermal method with its phase composition and morphology, and if the data are available, compare the antimicrobial performance of TiO_2_ samples with commercial TiO_2_ (Degussa P25). Discussion concerning fixed TiO_2_, i.e., deposited or incorporated on or into different materials, will be the subject of the following sections.

**Figure 9:**
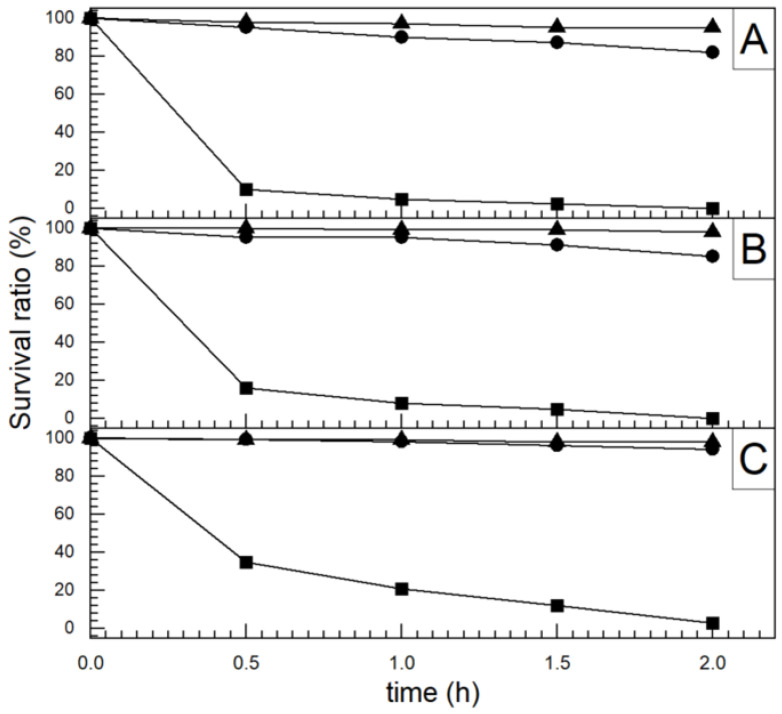
Effect of near-UV irradiation time at TiO_2_ (10 mg/mL) on the viability of bacteria: (**A**) *Salmonella choleraesuis*, (**B**) *Vibrio parahaemolyticus*, and (**C**) *Listeria monocytogenes* (dark (●), only UV light (▲), UV + TiO_2_ (■)) [108].

The antibacterial ability of the pure anatase phase was studied beginning from the first decade of this century, including commercial anatase [116], anatase prepared by conventional sol–gel [117,118] or the nonhydrolytic sol–gel method [119], and anatase prepared by the hydrothermal method [120]. All these studies indicated a pronounced ability of anatase to inactivate pathogens. Figure 9 shows a textbook example of time-kill curves for food-borne pathogenic bacteria (*Salmonella choleraesuis*, *Vibrio parahaemolyticus*, and *Listeria monocytogenes*) upon illumination of commercial anatase with a relatively small specific surface area (~3 m^2^/g) [116]. However, there is a discrepancy in the literature comparing anatase photoactivity with a standard reference catalyst, Degussa P25. For example, 30 nm-sized anatase nanoparticles prepared by sol–gel inactivate *E. coli* significantly faster than Degussa P25 [117]. On the contrary, Degussa P25 inactivates *E. coli* faster than the hydrothermally prepared anatase with a large specific surface area (~300 m^2^/g) [120]. The data discrepancy can result from differences in the properties of various samples, such as specific surface area, pore size distribution, number of hydroxyl groups on the surface, oxygen adsorption capacity, crystallite size, and crystal defects.

**Figure 10:**
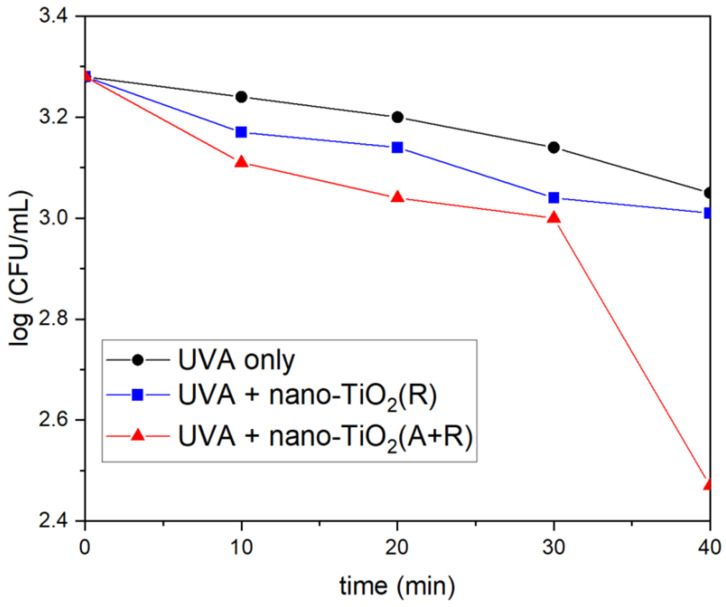
Kinetics of photocatalytic inactivation of *Bacillus anthracis* under the treatment of only UVA light and UVA light along with nano-TiO_2_ with a mixture of anatase and rutile phases (A+R) or pure rutile phase (R) [122].

The number of studies concerning the antibacterial activity of pure rutile phase is significantly smaller in comparison with anatase [121], with the prevailing opinion in the scientific community that rutile, as a high-temperature phase, is photocatalytically less active than anatase, most likely due to high-temperature processing followed by the formation of material with a lower specific surface area. An instructive example is a study by Prasad et al. [122] concerning photocatalytic inactivation of one of the most pathogenic microorganisms, *Bacillus anthracis*, using two commercial TiO_2_ nanopowders, a first mixture of anatase and rutile with an average size of ∼70 nm, and the second one with solely a rutile phase and an average size of ∼40 nm. Time-kill curves (Figure 10) undoubtedly show that TiO_2_ nanopowder with mixed phases inactivates pathogens more efficiently than the one with a pure rutile phase, even though its particle size is significantly smaller.

Since the anatase-to-rutile ratio in TiO_2_ samples, prepared by chemical methods, can be altered by adjusting calcination temperature and duration, the follow-up studies attempted to clear controversy about the antibacterial activity related to TiO_2_ with pure crystal phases (anatase or rutile) and Degussa P25 [123,124]. On one side, sol–gel prepared TiO_2_ samples, calcinated at 400, 600, and 800 °C, with pure anatase, mixed anatase/rutile, and pure rutile phase, respectively, display the antimicrobial activity in daylight on Gram-positive bacteria (*Staphylococcus aureus*, *Streptococcus pneumoniae*, and *Bacillus subtilis*), Gram-negative bacteria (*Proteus vulgaris*, *Pseudomonas aeruginosa*, and *Escherichia coli* [125]. However, the anatase sample has significantly higher antibacterial potency than the other two, as explained by the increase in crystallite sizes due to calcination at higher temperatures. On the other hand, Almashhori et al. [124] obtained TiO_2_ samples similar in size but with different anatase-to-rutile ratios using a microwave-assisted sol–gel technique. The most active sample against bacterial pathogens (*Escherichia coli*, *Klebsiella pneumoniae*, *Salmonella enterica*, *Pseudomonas aeruginosa*, *Proteus mirabilis*, *Enterococcus faecalis*, and *Staphylococcus aureus*) was the one with the highest ratio of anatase to rutile, i.e., the sample with a composition similar to Degussa P25 (81.65% anatase and 18.35% rutile phase).

A recent report by Yaemsunthorn et al. [125] provides an instructive study regarding the influence of TiO_2_ phase composition on photoactivity. They were able to synthesize the phase-tunable TiO_2_ nanoparticles under mild temperatures (up to 200 °C), producing two series of materials with different anatase-to-rutile ratios but otherwise similar morphologies. These authors employed hydroxylation of terephthalic acid (TA), the reaction used to identify HO^•^ radicals, to assess the phase-dependent photocatalytic oxidation capabilities of two series of materials having specific surface areas in the range of 70–120 and 20–57 m^2^/g.

**Figure 11:**
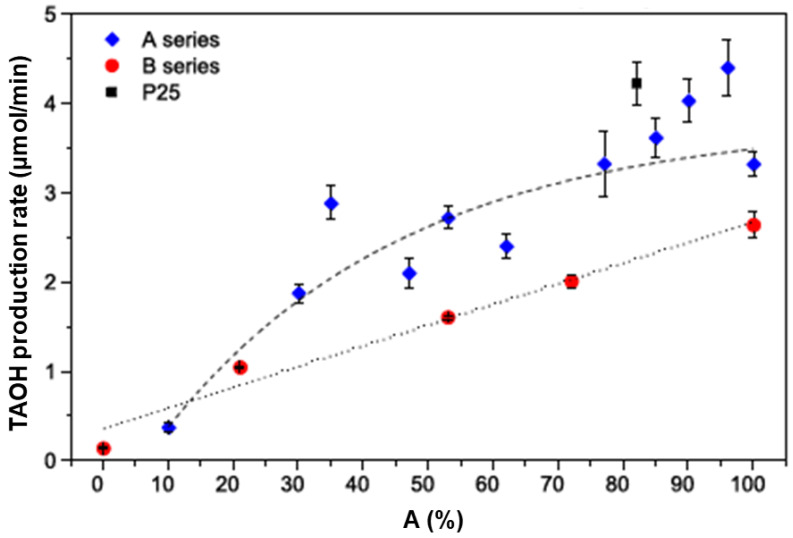
Photocatalytic oxidation-based hydroxylation of terephthalic acid (TA) over TiO_2_ as a function of anatase-to-rutile ratios (the specific surface area is within the range of: (◊) 70–120 m^2^/g (A series) and (○) 20–57 m^2^/g (B series); (■) Degussa P25 [125].

Figure 11 shows the reaction rate of hydroxylation of TA as a function of anatase-to-rutile ratios for two sets of samples and Degussa P25. We can conclude the following. First, the reaction kinetics become faster with the increase in the anatase-to-rutile ratio for both sets of samples. Second, smaller-sized TiO_2_ particles with higher specific surface (Series A) display higher photocatalytic ability than larger ones (Series B) with the same/similar phase composition. Third, the photocatalytic performances of the smaller-sized TiO_2_ samples with a high content of the anatase phase (≥80%) and Degussa P25 are comparable. Based on these observations, Yaemsunthorn et al. [125] concluded that photocatalytic reactions are most efficient with anatase-rich materials, in agreement with anatase-rutile band alignment in which rutile has a lower potential (higher energy) of the conduction band edge compared to anatase (Figure 7, right).

#### 4.2.3. Antibacterial Activity of TiO_2_ Prepared by Biological Methods

In recent years, due to growing awareness of the importance of ecologically friendly technologies, researchers have focused on replacing synthetic procedures, which include toxic precursors and high energy consumption, with safe, cost-effective, biocompatible, non-toxic, sustainable, and environmentally friendly biological methods. In biosynthetic methods, or so-called green synthesis, various biological resources available in nature, either plants (plant extracts) or microbial species (viruses, bacteria, fungi, algae), are one of the precursors for the synthesis of nanomaterials. In general, synthetic approaches using microorganisms do not have satisfactory repeatability, i.e., they do not provide stable nanomaterial morphology, including TiO_2_, nor the possibility for scaling up at the industrial level, compared to the synthesis using plant extracts. In addition, plants are more suited for the green synthesis of nanoparticles since they are non-pathogenic, so in this review, we will pay attention to their use for TiO_2_ synthesis.

The main components of plant extracts are polyphenols, including flavonoids, a significant class of polyphenols, and carboxylic acids. It is well-known that the most acidic phenolic hydrogen in polyphenols typically has a pKa in the pH range from 7 to 9 [126], and in slightly alkaline solutions, the hydroxyl group is a strong reductant for metal ions. As a result, phytosynthesis is now frequently used to prepare noble metal particles (Pt [127], Au [128], and Ag [129,130,131]) with the desired properties.

While in the scientific community, there exists a consensus that the hydroxyl group is a bioreductant for metal ions, on the other hand, the green synthesis of TiO_2_ is most frequently presented with a cartoon without any chemistry behind, followed by the conclusion that a change in extract color indicates the formation of TiO_2_ particles. In addition, in a limited number of studies concerning green synthesis of TiO_2_, different reaction mechanisms are proposed, even for the same precursors. Commonly used titanium precursors are titanium alkoxides (mainly titanium isopropoxide (TTIP)), titanium tetrachloride (TiCl_4_), metatitanic acid (H_2_TiO_3_), and titanium oxysulfate (TiOSO_4_), dissolved in distilled water or ethanol.

A few research groups believe that the extracted phytochemicals from plants in the formation process of TiO_2_ particles using TTIP act as capping agents, preventing TiO_2_ particle agglomeration, i.e., basically having the same role as commonly used capping agents, such as surfactants and polymers [132,133,134]. To put it bluntly, phytochemicals regulate the size of TiO_2_ particles by binding to the TiO_2_ surface over their functional groups without involvement in the hydrolytic reaction of TiO_2_ formation. However, there are opinions that, besides acting as capping and stabilizing agents in the hydrolysis of TiO_2_ precursors (for example, titanium (IV) butoxide), phytochemicals participate together with hydroxyl ions, forming intermediate complexes with deprotonated hydroxyl groups [135].

**Figure 12:**
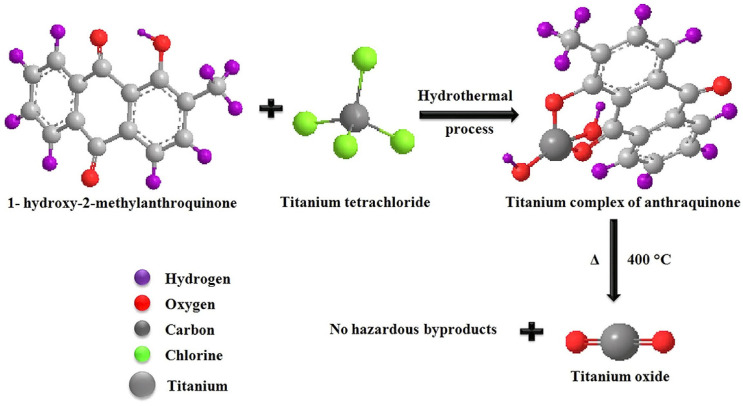
Proposed mechanism of green synthesis of TiO_2_ particles using as precursors TiCl_4_ and *M. citrifolia* leaves extract with anthraquinones as a main component [136].

Sundrarajan et al. [136] also proposed the involvement of phytochemicals in the reaction pathway of TiO_2_ formation when using, instead of alkoxides, TiCl_4_ as a source of titaniua. Figure 12 shows the coordination of 1-hydroxy-2-methylanthraquinone, the main component of *M. citrifolia* leaf extract, over carbonyl and hydroxyl groups in the intermediate complex with titanium, which transforms upon calcination to TiO_2_.

**Figure 13:**
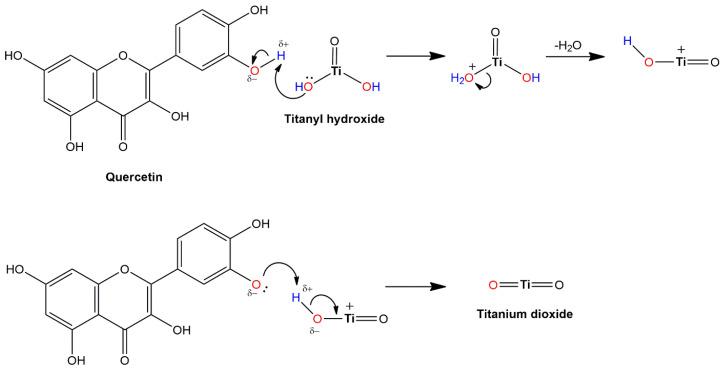
Possible reaction mechanism for the synthesis of TiO_2_ particles using as precursors titanyl hydroxide (TiO(OH)_2_), also known as metatitanic acid (H_2_TiO_3_), and quercetin, representative of the flavonoid group of polyphenols [140].

The reaction mechanism for the TiO_2_ formation using TiO(OH)_2_ [137,138,139,140,141] as a precursor differs from the reaction mechanism when titanium alkoxides or TiCl_4_ are the sources of titanium. As an example, the transformation of TiO(OH)_2_ to TiO_2_ using quercetin, a common and abundant flavonoid in plant extract, is presented in Figure 13. According to the literature, TiOSO_4_, a similar titanium source to TiO(OH)_2_, complies with this reaction mechanism [142,143]. In the first reaction step, due to the presence of lone electron pairs, the hydroxyl group from TiO(OH)_2_ picks up a hydrogen ion from the hydroxyl group of quercetin, forming an intermediate positively charged structure. Then, detachment of water from the positively charged intermediate takes place, resulting in a positively charged TiOOH intermediate, which, in a similar subsequent electron transfer process, results in the TiO_2_ formation.

Many research groups believe that the green synthesis of TiO_2_ is based on the ability of functional groups from phytochemicals to reduce titanium precursors (TiO(OH)_2_ [144,145], TiCl_4_ [146], TTIP [147]), indicated by the change in the solution color. However, when dissolved, titanium precursors do not exist as free Ti cations (Ti^4+^) but as complexes with water molecules or as part of the hydrolysis products. Whatever the mechanism is, the last preparation step, calcination, leads to the formation of TiO_2_ and the complete combustion of organic precursors. Of course, the organic scaffold directs the morphology and dispersibility of the final product. One more note concerning the synthesis. The color change of the extract is most likely a consequence of the interfacial charge transfer (ICT) complex formation [148,149] due to the condensation reaction between hydroxyl groups from phytochemicals and TiO_2_ surface hydroxyl groups, which we will discuss later on as one of the ways of improving the optical response of TiO_2_.

An overview of the literature data [136,141,142,143,147,150,151,152,153,154,155,156,157,158,159] on green-synthesized TiO_2_ employed against pathogens is summarized in Table 2. Table 2 also includes precursors (titanium source and plant extract) used for preparing TiO_2_ and the basic properties of the obtained material (structure and morphology). In contrast to studies concerning the antimicrobial activity of commercial TiO_2_ (Degussa P25), research on the biocidal performance of green-synthesized TiO_2_ is relatively recent, having started two decades ago, and is still ongoing. Based on the presented data, we can conclude the following. While the choice of titanium precursors is quite limited, the choice of biological precursors, on the other hand, is practically unlimited. The synthesized TiO_2_ nanoparticles were predominantly in the anatase crystal phase since the green synthetic approach does not necessarily require high thermal treatment. However, the appearance of the rutile crystal phase was also reported (see Table 2). In addition, the plant extract application provides reasonably good control of the TiO_2_ particle size and the possibility of preparing them in a nano-sized regime. Contrary to Degussa P25, where *E. coli* was a frequent choice for testing its antibacterial performance, various pathogen species were used to evaluate the antibacterial ability of green-synthesized TiO_2_ particles.

We direct readers to recent review articles [160,161,162] for additional information concerning diverse applications of green-synthesized TiO_2_ and other metal oxides.

### 4.3. Immobilized TiO_2_

Using suspended photocatalysts (TiO_2_ slurry) is challenging since it often leads to difficulties related to catalyst recovery from the reaction mixture and reusability, which may result in increased costs and environmental concerns associated with slurry disposal. To address these issues, the possibility of replacing suspended by immobilized photocatalysts on various solid supports was and is still under investigation, as emphasized in recent review papers [163,164,165]. In addition, TiO_2_ immobilization is a flexible process, providing the possibility to fix particles onto chosen substrates or matrices for a specific application. Although immobilization of photocatalysts frequently reduces their performance, the transition from suspended to immobilized photocatalytic systems is a significant advancement in this field.

Immobilized TiO_2_ on solid supports can exist as individual/agglomerated bound particles or thin films. Of course, the photoinduced activity of thin films is often lower due to a decrease in the surface-to-volume ratio. On the one hand, this review will cover TiO_2_-based nanocomposites with polymers, natural or artificial, which have a wide variety of applications, including food packaging, antimicrobial, and self-cleaning textile materials. On the other side, thin TiO_2_ films on common substrates such as glass and metals provide, for example, long-lasting protection against pathogens in hospitals or medical devices, respectively. Immobilization of TiO_2_ particles or thin film formation on solid supports is achievable using previously prepared TiO_2_ particles/powders or by synthesis, mainly using the sol–gel method.

#### 4.3.1. Antibacterial Activity of Thin TiO_2_ Films

The presence of thin TiO_2_ films brings additional functionality and, consequently, additional value to the support. Of course, the nature of the supports and their applications governs the methods of thin TiO_2_ film deposition. For example, to decrease the probability of infection, especially in hospitals, schools, restaurants, industrial facilities, public places, and playrooms, the antimicrobial activity of TiO_2_ films on fiber-cement surfaces [30] and TiO_2_ additives present in the glaze of ceramic tiles [164] was studied. Both applications imply large-scale production, so cost-effective, commercial TiO_2_ is a suitable material. In addition, simple deposition or incorporation techniques, such as the doctor blade method for TiO_2_ film deposition [30] or the incorporation of TiO_2_ in the reaction mixture for industrial production of ceramic tiles [166], are recommended.

**Figure 14:**
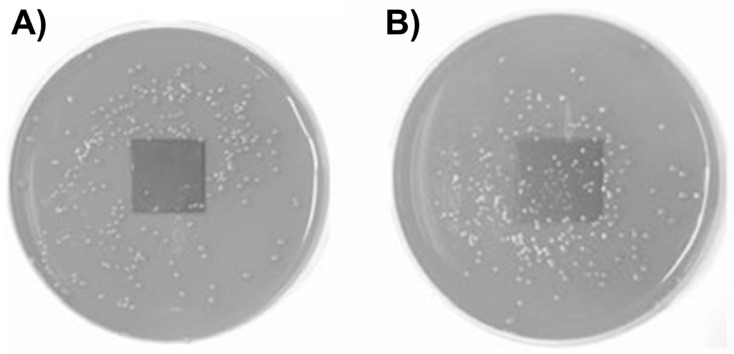
The *E. coli* colonies above metal plates (**A**) coated with TiO_2_ and (**B**) without TiO_2_ coating illuminated with UV light [167].

For sophisticated applications, such as orthopedic implants, the role of TiO_2_ coatings is to improve the implant’s compatibility with tissue and resistance to microbial colonization on biomaterial surfaces. Naturally, methods of coating metallic surfaces with TiO_2_ are more complicated than those described above. An instructive example is a study by Tsuang et al. [167]. These authors applied the dip-coating method to deposit thin TiO_2_ films on stainless steel using previously prepared colloids consisting of nanometer-sized TiO_2_ particles as precursors. Figure 14 displays the bactericidal effect of TiO_2_ nanoparticle-coated metal plate on the bacterial colonies of *Escherichia coli*. The formation of *E. coli* colonies is inhibited significantly in the presence of a coated metal plate with TiO_2_ under UV light excitation (Figure 14A). On the other hand, in the control experiments, *E. coli* survived well upon UV light illumination when metal plates did not have a TiO_2_ coating (Figure 14B). Currently, titanium implants, due to their excellent mechanical properties and biocompatibility with human tissues, are of particular scientific interest. Jia et al. [168] fabricated TiO_2_ nanorods on the surface of pure titanium by using hydrothermal and annealing methodology. They demonstrated that the prepared samples display efficient photocatalytic antifungal and antibacterial ability against *C. albicans*, *A. actinomycetemcomitans*, and *P. gingivalis*.

Dip-coating is a versatile technique, and besides metal surfaces, TiO_2_ can be deposited on various substrates. For instance, Ag-doped TiO_2_ thin films prepared via dip-coating on glass substrates can be used for wastewater treatment, where they photocatalytically degrade organic dyes (e.g., methylene blue) and demonstrate improved performance over pure TiO_2_ films due to enhanced generation of reactive oxygen species and reduced electron–hole recombination [169].

**Figure 15:**
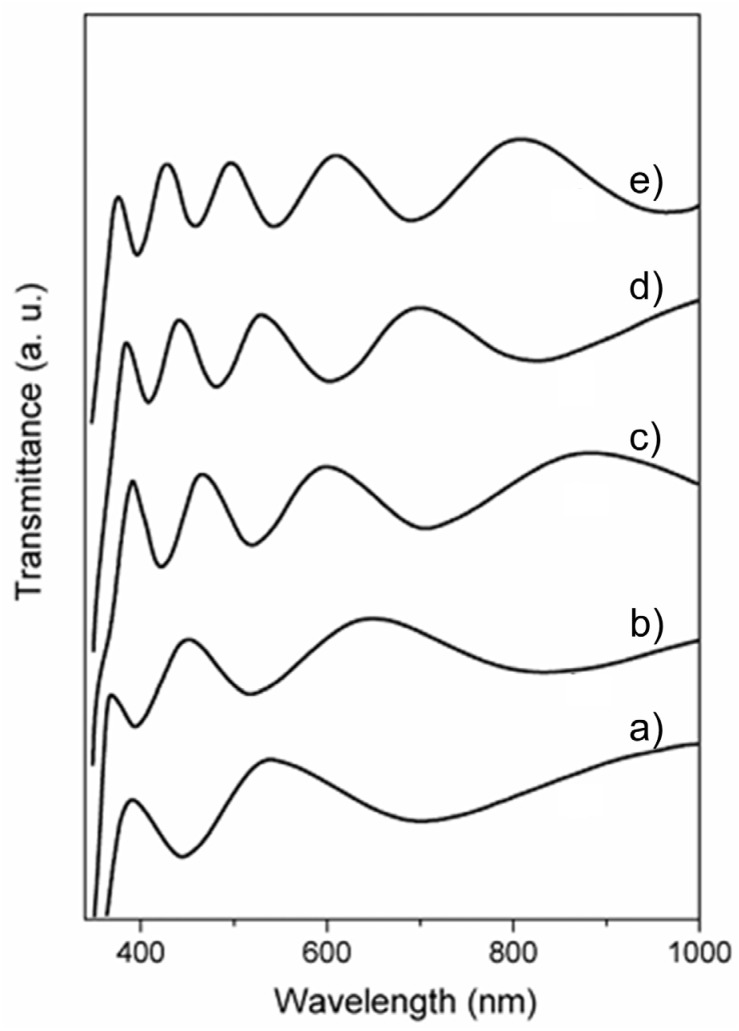
Transmission spectra of nanocrystalline TiO_2_ films on glass slides as a function of number of depositions: (**a**) 3, (**b**) 4, (**c**) 5, (**d**) 6, and (**e**) 7. Adapted from [170].

For fundamental research, it is convenient to use transparent supports, such as glass slides [170]. Besides the similar nature of TiO_2_ (coat) and silica (support), an additional benefit is the possibility of using a simple spectrophotometric method [171] to control film thickness during the dip-coating process. Figure 15 displays the transmission spectra of TiO_2_ films on glass slides prepared by the dip-coating technique as a function of the number of depositions [172]. The thickness of TiO_2_ films can be calculated from the position of interference fringes in transmission spectra [171]. Briefly, locations of the extrema (maxima and minima) in the transmission curve are uniquely determined by the product nd:

(13) $$\lambda_{ext}=\;{\left(\frac{m}{4nd}\right)}^{-1}\;m=1,\;2,\;3,\;\ldots$$

where m is the order of extremum from an arbitrary reference extremum, n is the refractive index, and d is the thin film thickness. Plotting 1/λ_ext_ against m (the extremum order) results in a straight line with a 1/4nd slope. Therefore, knowing the value of the refractive index (n), it is simple to calculate the thickness of each deposited film and the growth with each deposition. The increase in thickness of nanocrystalline anatase films in the specific case shown in Figure 15 was calculated to be around 80 nm per deposition [170]; the anatase refractive index is 2.524.

**Figure 16:**
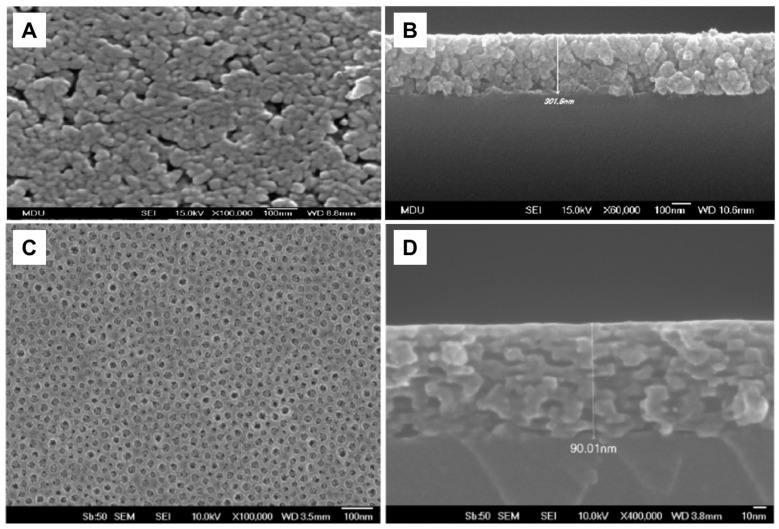
SEM images of TiO_2_ films on supports prepared by deep-coating technique in the absence (**A**,**B**) and the presence of polymer template (**C**,**D**); top view: (**A**,**C**); cross-section: (**B**,**D**) [172,173].

Similarly to the dip-coating method for thin film fabrication is the spin-coating method. The advantage of spin-coating over the dip-coating technique is better control of film thickness and its uniformity, while the disadvantage is a significant waste of material. So, the dip-coating method is more suitable for coating large objects with complex shapes. Figure 16A and Figure 16B display SEM images of a top view and cross-section, respectively, of TiO_2_ film prepared by deposition of nanoparticles synthesized by sol–gel using the spin-coating method [172]. With knowledge of the importance of the specific surface area in photocatalytic processes, including pathogen inactivation, attempts were made to prepare mesoporous TiO_2_ films using the dip-coating method and a solution consisting of TiO_2_ nanoparticles and polymer, serving as a template [173]. Opposite to coatings prepared using TiO_2_ colloids without polymer templates (Figure 16A,B), the top view of the TiO_2_ film, prepared in the presence of a polymer template, indicates a complete crack-free coverage of the substrate and noticeable porosity (Figure 16C). In addition, the SEM image of the cross-section (Figure 16D) revealed a short-range, cubic-like ordering of the interconnected pore structure.

Since the sphere has the smallest surface area for a given volume, another way to increase the specific surface area is to prepare and deposit elongated particles on the substrate. Initiated by the pioneering study by Assefpour-Dezfuly et al. [174], the scientific community paid enormous attention to titania nanotube arrays fabricated by anodic oxidation of titanium foil, which resulted in hundreds of papers per year over the last few decades, frequently being summarized in review articles such as Fu et al. [175]. However, although the synthesis of TiO_2_ nanotube arrays is a well-developed area, the suitable technology for large-scale industrial production with precise control of nanotube geometry has yet to be further investigated.

**Figure 17:**
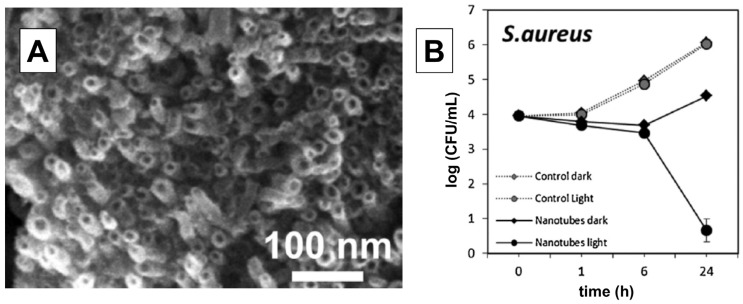
(**A**) SEM image of tightly bound TiO_2_ nanotubes; top view. (**B**) Antibacterial activity of TiO_2_ nanotubes against *S. aureus* as a function of time; Müeller-Hinton broth inoculated with *S. aureus* served as a control [177].

Besides as-prepared TiO_2_ nanotubes [176,177], the antibacterial activity of loaded TiO_2_ nanotubes with antibiotics [178] was tested mainly using Gram-negative bacteria (*E. coli* [177,178] and *K. pneumoniae* [178]) and Gram-positive (*S. aureus* [177,178] and *B. atrophaeus* [176]) bacteria. The enhanced ability of TiO_2_ nanotubes to inactivate pathogens compared to commercial Degussa P25 is well-documented in a study by Podporska-Carroll et al. [177]. Figure 17A shows an SEM image of the top view of the tightly packed TiO_2_ nanotubes within bundles with an average outer and inner diameter of approximately 20 and 8 nm, respectively, and consequently a high specific surface area (150 m^2^/g). TiO_2_ nanotubes, exposed to UV light, showed excellent antibacterial activity against *S. aureus* (Figure 17B). However, the same amount of commercial TiO_2_ (Degussa P25), illuminated for the same time as the TiO_2_ nanotubes, did not exhibit antibacterial properties. So, Podporska-Carroll et al. [177] concluded that antibacterial activity, besides material properties (optical response, specific surface area, aspect ratio, interface), depends significantly on the experimental conditions of the biological investigations.

#### 4.3.2. Antibacterial Activity of TiO_2_–Polymer Nanocomposites

The development of antimicrobial materials by the deposition or incorporation of biocidal agents, in this case TiO_2_, onto or within a polymer support or matrix is challenging because it is difficult to obtain homogeneously dispersed hydrophilic nanoparticles onto or within a hydrophobic polymer [179]. Although many research groups have made an effort to prepare polymer-based nanocomposites with TiO_2_, the number of review articles is small. The review articles by Montazer et al. [180] and Radetić [181], published at the beginning of the second decade of this century, accompanied a recent review by Rashid et al. [179]. For clarity reasons, we will cover in this review advanced textile materials and food-packaging materials, which represent typical antibacterial applications of two types of polymer nanocomposites formed either by deposition or incorporation of TiO_2_ onto or within the polymer, providing UV protection as an additional benefit [182,183,184]. However, the cautious choice of polymer is necessary to avoid its oxidation, followed by degradation, induced by the photocatalytic action of TiO_2_.

**Figure 18:**
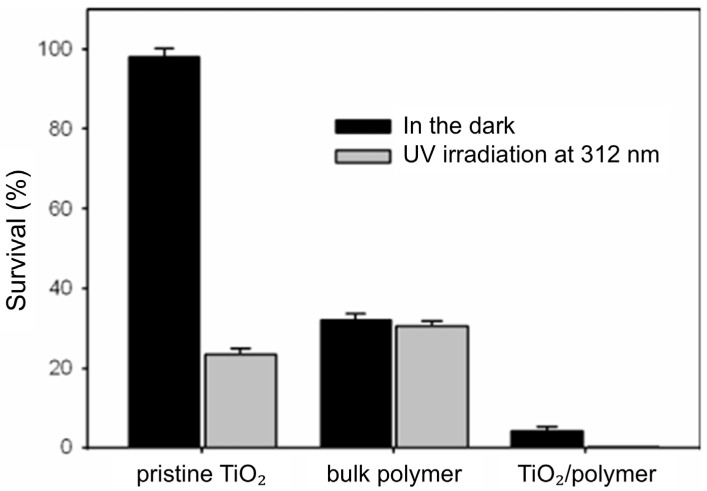
Percent of *S. aureus* survival after treatment with pristine TiO_2_, bulk poly [2-(*tert*-butylamino)ethyl methacrylate-*co*-ethylene glycol dimethacrylate] and core–shell TiO_2_-poly [2-(*tert*-butylamino)ethyl methacrylate-*co*-ethylene glycol dimethacrylate] particles in the absence and the presence of UV light [185].

However, TiO_2_ excited by UV light can induce the polymerization of monomers adsorbed on its surface, which results in the formation of core–shell particles. TiO_2_ particles functionalized with polymers can be used as an antibacterial agent or can be further processed as building blocks to synthesize nanocomposites. A helpful illustration of the synergistic antibacterial effect of constituents in core–shell TiO_2_-poly [2-(tert-butylamino)ethyl methacrylate-co-ethylene glycol dimethacrylate] particles, abbreviated as TiO_2_-poly(TBAM-co-EGDMA), generated photocatalytically, is the work by Kong et al. [185]. Figure 18 shows the inactivation of *S. aureus* when exposed to core–shell TiO_2_-poly (TBAM-co-EGDMA) particles and their constituents, pristine TiO_2_ and bulk poly(TBAM-co-EGDMA), either in the dark or when aroused by UV light. The core–shell TiO_2_-poly(TBAM-co-EGDMA) particles display better antibacterial abilities than pure TiO_2_ particles and the bulk polymer in the presence and absence of UV light.

Immersion of textiles, natural and chemical, in previously prepared TiO_2_ colloids, followed by drying, is the standard procedure for textile functionalization. However, to increase attraction between hydrophilic particles and hydrophobic textile surfaces, and consequently to obtain homogeneously distributed TiO_2_ particles across the textile surfaces, either the textile surface should be pretreated or the surface of TiO_2_ particles modified. Plasma treatment [186,187,188] and sonochemical techniques [189] are textile pretreatment processes frequently applied to increase the roughness and change the chemical composition of textile surfaces, introducing polar groups such as hydroxyl and carboxyl.

Noteworthy are the back-to-back papers from the Serbian team concerning the TiO_2_ deposition on plasma-activated polyester [190,191,192] and cotton [193] fabrics. Table 3 summarizes the relative intensity of high-resolution C1 photoelectron peaks, corresponding to carbon atoms in the aromatic ring (C–C, C–H), methylene carbons singly bound to oxygen (C–O), and ester carbon atoms (O–C=O) for the untreated polyester (U-PES) and oxygen and argon plasma-treated polyesters (O_2_-PES, and Ar-PES) fibers [190]. It is evident that plasma treatment of polyester, independently of the applied gas, caused the appearance of the C=O groups and the increase in the percentage of C–O groups at the expense of C–C, C–H, and O–C=O groups. The plasma-treated polyesters are more accessible to hydrophilic colloidal TiO_2_ nanoparticles due to increased hydrophilicity and can form stable nanocomposites accommodating several times higher TiO_2_ content than the untreated ones.

**Figure 19:**
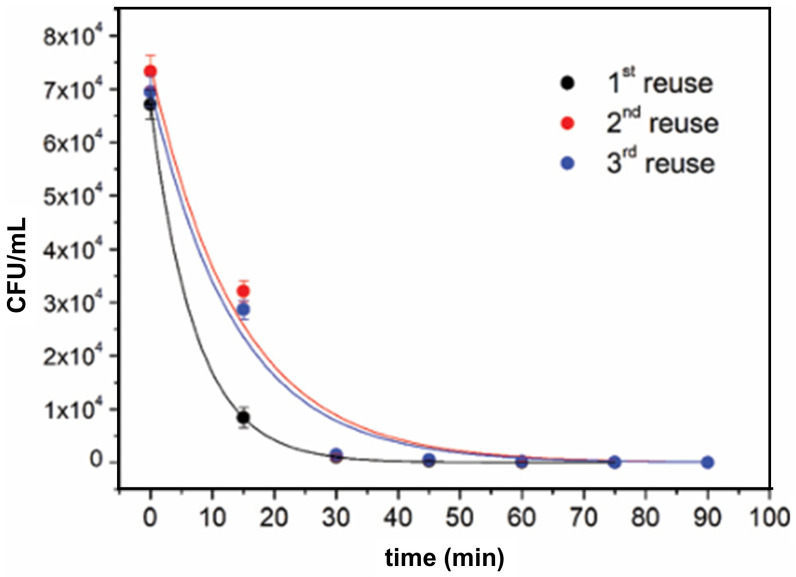
The photocatalytic inactivation kinetics of *E. coli* over TiO_2_-cellulose nanocomposite in recycling experiments. Adapted from [194].

The reusability studies of photocatalytic materials are crucial for assessing their potential for large-scale applications. The effect of washing at a temperature of 90 °C for 60 min before any subsequent tests on the antimicrobial performance of TiO_2_ deposited on a cellulose surface by the plasma sputtering technique is shown in Figure 19 [194]. The time-kill kinetic curves of *E. coli* (Figure 19) indicate satisfactory reusability of TiO_2_-cellulose nanocomposite, since bacterial inactivation capacity after the second and third regeneration processes is very close to the inactivation capacity of the as-prepared nanocomposite. The only difference in the inactivation kinetics of *E. coli* between reused and first-time used TiO_2_-cellulose fabrics is observable in the first few minutes of photocatalytic experiments, in which the bacterial inactivation performed by the reused fabrics is slower. The detachment of TiO_2_ particles from the cellulose surface during the washing process causes a slight decrease in bacterial inactivation capacity.

In some cases, the use of sophisticated plasma pre-treatment of textile surfaces or complexed chemical functionalization of TiO_2_ surfaces is not necessary to obtain high-quality, durable antimicrobial coatings. For example, the TiO_2_-cotton nanocomposites, prepared by taking advantage of the interaction between positively charged TiO_2_ surface hydroxyl groups and the negatively charged hydroxyl groups on cellulose fibers obtained by pretreatment with NaOH, display high antibacterial efficiency against Gram-negative and Gram-positive bacteria (*E. coli* and *S. aureus*, respectively) [195].

Another simple approach to enhance the deposition of biocidal agents, nanometer-sized particles, onto fabrics is the use of green materials, naturally occurring polysaccharides (alginate and chitosan), as textile fiber modifiers. Functionalized polyester fabrics with alginates and TiO_2_ nanoparticles exhibit outstanding antibacterial activity against *E. coli* and UV protection efficiency even after five washing cycles, indicating excellent laundering durability [196]. The cotton-chitosan-TiO_2_ nanocomposites, apart from the intrinsic antibacterial character of chitosan, also display the high reduction rates of *E. coli* and *S. aureus*, and in addition, enhanced UV-protection properties [197].

**Figure 20:**
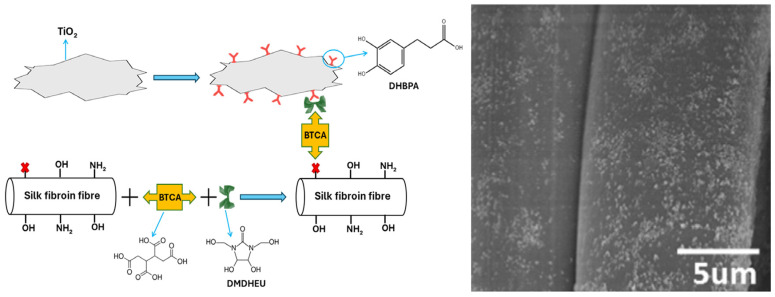
Schematic illustration of the synthetic procedure to covalently bind TiO_2_ particles to the silk surface; SEM image of prepared TiO_2_-silk nanocomposite using the described synthetic approach [200].

Examples of functionalized TiO_2_ particles, prepared by sol–gel and hydrothermal methods utilizing tetrabutyl titanate and amino polymers as precursors [198], attached to cotton textiles are apatite-coated and amino-capped TiO_2_ nanoparticles [199]. An excellent study of the intricate interface chemistry that results in the production of covalently attached TiO_2_ particles on the silk surface is a work by Li et al. [200]. The synthetic procedure has three steps, as shown in Figure 20. On one side, surface-modified TiO_2_ is created using 3-(3,4-dihydroxyphenyl) propionic acid (DHBPA), a natural antiviral compound; on the other side, silk is grafted with dimethyloldihydroxyethyleneurea (DMDHEU) over 1,2,3,4-butanetetracarboxylic acid (BTCA) as a bridge crosslinker. The first and second synthetic steps are separate processes. The last third phase involves the formation of a covalent link between the inorganic and organic components of the nanocomposites by a reaction between the pendant carboxylic acid groups of DHBPA and the hydroxyl groups of DMDHEU. The silk surface exhibits a uniform distribution of TiO_2_ particles, as seen in the SEM image (Figure 20).

TiO_2_-polymer nanocomposites, exerting environmentally friendly antimicrobial activity without releasing potentially toxic materials into the surrounding media, even against strains resistant to multiple drugs, have been attracting the attention of the research community for potential applications in the food industry since the beginning of this century. However, the synthesis of polymer-based nanomaterials for food packaging applications, besides compatibility of inorganic and organic components, the main issue in fabrication of smart textiles, faces considerable additional challenges regarding mechanical (strength, Young’s modulus, elongation at break) and thermal properties (glass transition temperature, melting temperature), permeability of gasses (oxygen transmission rate (OTR), water vapor permission), gas and microbial sensing ability for the food quality monitoring, and biodegradability, as pointed out in highly cited review articles by Duncan [201], several recent ones [202,203,204], and those published this and previous year [205,206,207]. Of course, incorporating metal oxides, such as TiO_2_, into a polymer matrix protects food from exposure to UV light, thereby increasing its shelf life [208].

Targeted polymer materials for food packaging applications are water-soluble polymers, such as the promising biodegradable material polyvinyl alcohol (PVA) [208], polymers with limited solubility in water but soluble in alcohol-water mixtures and some polar aprotic solvents (for example, ethylene-vinyl alcohol copolymers (EVOH)) [209], and hydrophobic polymers, soluble in organic solvents, such as low-density polyethylene (LDPE) [210,211,212], oriented-polypropylene (OPP) [213], and poly(lactic acid) (PLA) [214].

**Figure 21:**
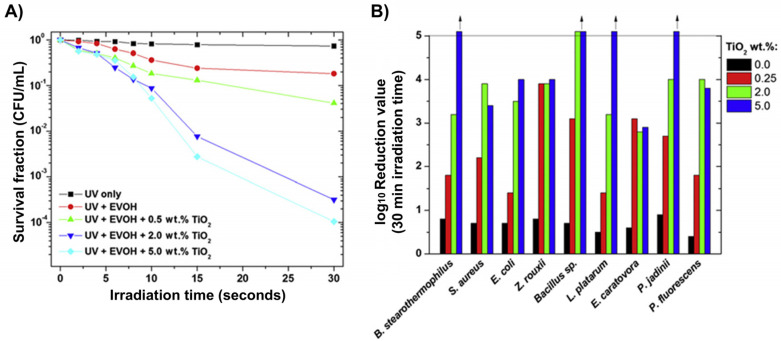
(**A**) Survival fraction of *E. coli* as a function of concentration of incorporated TiO_2_ in the ethylene-vinyl alcohol (EVOH) and UV illumination time. (**B**) Total logarithmic reduction in numerous food-relevant microorganisms after 30 min of illumination time in the presence of the TiO_2_-EVOH composite materials. Bars with upward-pointing arrows represent samples where the log-reduction was greater than 5 [201].

Early work by Cerrada et al. [215] provides a model for investigating the potential ability of TiO_2_-polymer composites for food packaging applications. These authors studied the photocatalytic inactivation of nine microorganisms (bacteria and yeasts), frequently involved in food poisoning and spoilage, over TiO_2_-EVOH films excited by UV light. Figure 21A shows the dependence of *E. coli* inactivation kinetics on the weight percentage of incorporated TiO_2_ in EVOH. On the other hand, Figure 21B displays a comparison of the reduction in nine food-relevant microorganisms over TiO_2_-EVOH films with different compositions after 30 min of UV illumination. Another instructive example is a technologically oriented study by Chawengkijwanich et al. [213], which proved the ability of polypropylene films coated with TiO_2_ nanoparticles to inhibit *E. coli* growth on fresh-cut lettuce under commercial light sources (fluorescent and black-light bulbs).

### 4.4. TiO_2_—Noble Metal Heterostructures

**Figure 22:**
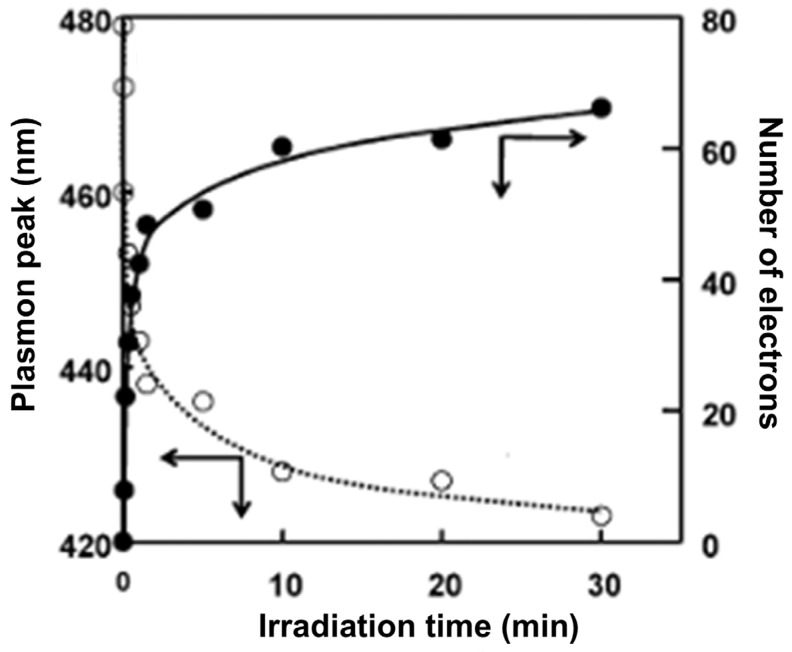
The peak position of plasmon resonance band in core–shell Ag@TiO_2_ nanoparticles as a function of number of stored electrons, i.e., time of UV irradiation [216].

The major drawback of TiO_2_-based photocatalysts, besides the limited light absorption in the UV spectral range (<390 nm), is the rapid recombination of photogenerated charge carriers (electron-hole pairs), which leads to the annihilation of charges rather than their migration to the catalyst surface and subsequent reactions with adsorbed materials. The formation of heterostructures between TiO_2_ and noble metals is a proven strategy to suppress the recombination rate of photogenerated electron-hole pairs due to their enhanced separation. The enhancement of the charge separation process is a consequence of energy alignments in TiO_2_-based heterostructures [215]. Since the conduction band of TiO_2_ is higher than the Fermi level of any of the noble metals, photogenerated electrons are transferred from TiO_2_ to the metal, forming a space charge layer whose electric field drives electrons into the interior of metallic particles. Consequently, the larger amount of photogenerated holes can reach the TiO_2_ surface and produce the reactive species, which further facilitate the photocatalytic oxidation process, such as inactivation of pathogens. The optical properties of noble metal particles, besides size and shape, are dependent on the electron density. The charge separation process can be indirectly visually observed and easily followed spectroscopically, as demonstrated by Hirakawa and Kamat [216], who provided the peak-dependent position of the plasmon resonance band as a function of the number of stored electrons in core–shell Ag@TiO_2_ nanoparticles (Figure 22). Also, the charging and discharging of colloidal Ag nanoparticles prepared using NaBH_4_ as a reducing agent, accompanied by oscillations in the plasmon resonance band peak position, were observed as a function of aging time due to metal-catalyzed hydrolysis of excess NaBH_4_ [217].

Generally, the heterostructures between TiO_2_ and noble metal nanoparticles (Ag [200,218,219,220,221,222,223,224,225,226,227,228,229,230,231,232,233,234,235,236,237,238,239,240,241,242,243,244], Au [245,246,247,248], Pt [240,248,249], and Pd [250]) can be prepared by two different methods: impregnation, combining separately prepared TiO_2_ and noble metal particles, and photodeposition, taking advantage of the above-mentioned favorable energy alignment, which provides an opportunity for photocatalytic reduction in ionic or ligand-coordinated noble metal species to metallic particles by TiO_2_. The use of bidentate ligands capable of simultaneously covalently binding to the TiO_2_ surface and chelating metal ions present in the surrounding solvent is a prerequisite for the efficient photocatalytic fabrication of TiO_2_–noble metal heterostructures [251]. Although not directly correlated to this subject, to illustrate the capability of this approach, we emphasize the photocatalytic reduction of cadmium ions to metallic cadmium over surface-modified TiO_2_ nanoparticles with aromatic and aliphatic amino acids (histidine and alanine, respectively), which is a thermodynamically unfavorable process with unmodified TiO_2_ nanoparticles [252].

**Figure 23:**
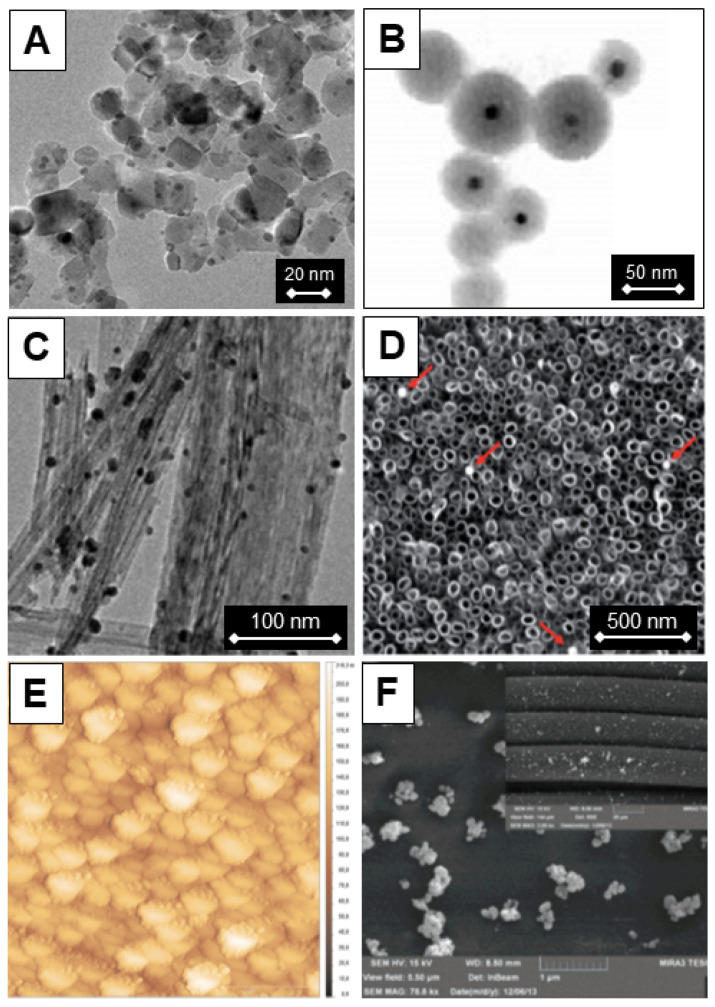
Microscopic images of several TiO_2_-Ag composites: (**A**) silver-decorated commercial Degussa P25, (**B**) core–shell Ag@TiO_2_ nanoparticles, (**C**) free-standing titanate nanotubes decorated with Ag nanoparticles, (**D**) silver-decorated TiO_2_ nanotubes on titanium support (arrows indicate the Ag nanoparticles), (**E**) silver particles on the nanocrystalline TiO_2_ film, and (**F**) polyamide fabric impregnated with TiO_2_-Ag nanoparticles [99,216,232].

A literature overview showed that various architectures of TiO_2_-Ag heterostructures are the most extensively studied antimicrobial TiO_2_-based heterostructures incorporating noble metal particles. Figure 23 displays microscopy images of different morphological forms of TiO_2_ (commercial Degussa P25, spheres, and tubular particles), free or attached to the support, decorated with Ag nanoparticles, or having a core–shell architecture. We prepared Figure 23 by combining the microscopy data from references [99,216,232,234,242].

**Figure 24:**
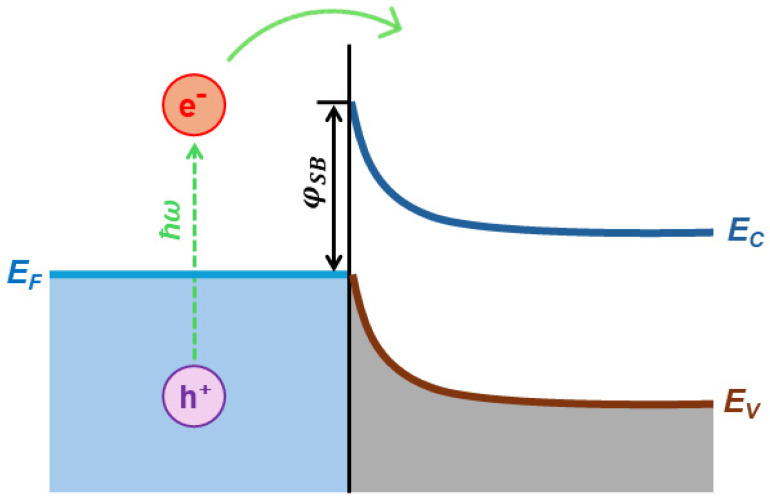
The charge-transfer steps in TiO_2_-based heterostructures with noble metal nanoparticles under Vis light excitation. (E_F_—Fermi level, E_C_—Conduction band edge, E_V_—Valence band edge).

Besides acting as electron traps by promoting electron-hole separation, the noble metal nanoparticles (Ag and Au) absorb visible light due to their surface plasmon resonance, thus forming visible light-driven photocatalysts when combined with TiO_2_. Although we dedicated the following section to the antimicrobial activity of TiO_2_-based visible-light-responsive composites, an explanation of the catalytic activity of TiO_2_–noble metal particle heterostructures under visible light excitation is provided in this section [246,253,254]. Figure 24 depicts the possible electron generation and subsequent electron transfer from noble metal particles to TiO_2_. The visible light absorption resulting from the direct excitation of the surface plasmon resonance band of noble metal (NM) leads to the generation of hot electrons ${(e}_{hot}^{-}$). Hot electrons with sufficient energy can overcome a Schottky barrier between a plasmonic particle and a semiconductor and inject, in this case, into the conduction band of TiO_2_ $(e_{CB}^{-}$) on a femtosecond time scale. The Schottky barrier is labeled as $\varphi_{SB}$ in Figure 24. Of course, electrons in the conduction band of TiO_2_ get trapped ${(e}_{tr}^{-}$) and further react with oxygen, while the positive holes oxidize substrates present on the surface [99,216,232]. For clarity, the formation of reactive species induced by the visible light excitation of TiO_2_-based heterostructures with noble metal particles is given by the following equation:

(14) $$NM\overset{h\nu }{\to }e_{hot}^{-}\to e_{CB}^{-}\to e_{tr}^{-}\overset{O_{2}}{\to }O_{2}^{-•}$$

However, the rapid back electron transfer and consequent charge recombination limit the efficiency of the photocatalytic processes. In addition, the generation and electron transfer from noble metal nanoparticles to TiO_2_ strongly depend on the height of the Schottky barrier and optical properties of noble metal particles, i.e., their size and shape. In particular, it is well-known that the optical properties of elongated noble metal nanoparticles are sensitive to their aspect ratio, as shown in the review article by Pérez-Juste et al. [255].

Corrosion or dissolution of the noble metal particles occurs during the photoinduced catalytic reactions. This effect is the most pronounced for Ag particles since silver is the least noble among all noble metal particles. While dissolution of noble metal particles deposited on TiO_2_ generally limits their use in photocatalytic processes, for example, in photocatalytic degradation of organic pollutants, the toxic action of silver is known from ancient times, and prevailing opinion is that silver’s biocidal effects originate from released silver ions during the course of silver dissolution, as we mentioned in the Introduction. Since silver, or better to say Ag^+^ ions, has a pronounced antimicrobial ability, and the deposition of Ag particles on the TiO_2_ surface improves the optical response of the heterostructure and charge separation of photogenerated electron-hole pairs, this answers why most of the studies concerning the antimicrobial performance of TiO_2_-based heterostructures with noble metal particles are actually about the TiO_2_-Ag heterostructure [218,219,220,221,222,223,224,225,226,227,228,229,230,231,232,233,234,235,236,237,238,239,240,241,242,243,244,245,246,247,248,249,250,251]. In addition, there is a synergism between Ag and TiO_2_ since Ag^+^ ions are enhancing the generation of ROS, as pointed out by Foster et al. [70] and illustrated by the following set of equations:

(15) $${Ag}^{+}+O_{2}^{-•}\to {Ag}^{0}+O_{2}$$

(16) $${Ag}^{0}+O_{2}^{-•}\to {Ag}^{+}+O_{2}^{2-}$$

(17) $$H_{2}O_{2}+{Ag}^{0}\to HO^{-}+HO^{•}+{Ag}^{+}$$

To put it bluntly, the superoxide radical anion, formed by reducing oxygen by photogenerated electrons in TiO_2_, is transformed to the most powerful oxidizing species, the hydroxyl radical, when silver switches between the +1 and zero states.

Based on the literature data in this area [218,219,220,221,222,223,224,225,226,227,228,229,230,231,232,233,234,235,236,237,238,239,240,241,242,243,244,245,246,247,248,249,250], it is evident that TiO_2_-based heterostructures containing noble metal particles exhibit a greater ability to inactivate microbes compared to TiO_2_ alone. However, determining to what extent the improved antimicrobial performance in heterostructures is a consequence of enlarged charge separation of electron-hole pairs, or improved light-harvesting ability due to surface plasmon resonance absorption, or dissolution of metal particles and release of metal ions in the surrounding media, which is especially significant in the case of TiO_2_-Ag heterostructures, is difficult. So, further, we will briefly discuss a few papers that have attempted to address these issues and direct readers to a large number of original studies.

M. Sökmen et al. [218] compared the photocatalytic ability of commercial TiO_2_, entirely with anatase structure, and TiO_2_-Ag composites, loaded with silver up to 1 wt.-%, to inactivate *E. coli* under UV light illumination. Table 4 collects the data concerning surviving *E. coli* colonies as a function of illumination time for both photocatalysts, neat and Ag-loaded TiO_2_. While an illumination period of 15 min was necessary for complete *E. coli* inactivation with neat TiO_2_, the inactivation with TiO_2_-Ag composite was so efficient that all *E. coli* cells were inactivated immediately when combined with the TiO_2_-Ag photocatalyst. The sharp decline in CFU observed between 15 and 20 min for TiO_2_ reflects a typical photocatalytic inactivation pattern, where ROS accumulation during the initial phase is followed by rapid cell wall penetration and sudden loss of bacterial viability once a critical ROS threshold is reached. In addition, the Ag content in TiO_2_-Ag composite is only 1 wt.-%, and efficient inactivation of *E. coli* was observed even for concentrations as low as 0.1 mg/mL.

**Figure 25:**
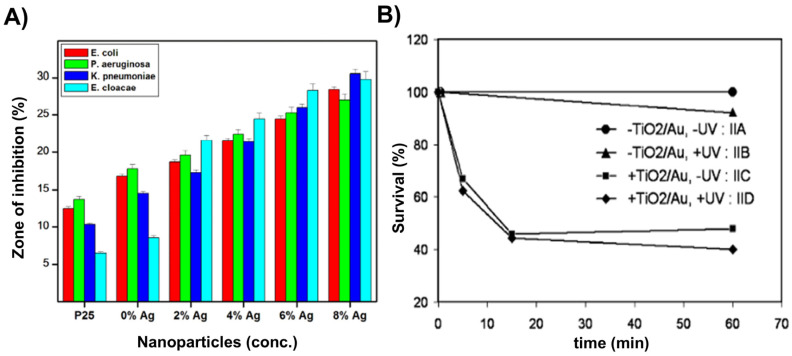
(**A**) Bar graph for zone inhibition assays against *E. coli*, *P. aeruginosa*, *K. pneumoniae*, and *E. cloacae* of commercial Degussa P25 powder, as-prepared TiO_2_ particles, and Ag-loaded TiO_2_ with different contents of silver. (**B**) Changes in the survival of *E. coli* (~10^5^ CFU/mL) in the absence (a and b) and in the presence of TiO_2_-Au coatings on glass slides under room light and low-power LED device emitting in UV (c and d); control experiments: (●), (▲), (■), and (♦) are a, b, c, and d, respectively [242,245].

On the other hand, Ali et al. [236] compared the antimicrobial activity of Degussa P25, non-spherical anatase TiO_2_ particles with an average size of about 10 nm synthesized by the sol–gel method, and Ti_1−x_Ag_x_O_2_ (0.00 < x < 0.08) composites by the disc diffusion method against Gram-negative bacteria (*E. coli*, *P. aeruginosa*, and *K. pneumoniae*) under visible light illumination. Figure 25A shows the percentage of zone of inhibition for different bacterial strains *versus* Degussa P25, as-prepared TiO_2_ particles, and TiO_2_-Ag composites with increasing silver content from 2 to 8 mol%. The commercial Degussa P25 TiO_2_ shows a very narrow inhibition zone for all bacterial strains, even compared to as-prepared pure TiO_2_. For TiO_2_-Ag composites, enhancement of bactericidal activity against all studied bacterial strains as a function of the silver content, indicated by enlargement of the inhibition zone, is a consequence of the inherent antimicrobial property of silver. The significance of silver is, in addition, supported by the fact that the inhibition zone for all bacterial strains in TiO_2_-Ag composite with the highest silver concentration (8 mol%) reached almost twice the values of the as-prepared pure TiO_2_.

For many applications, particularly those in indoor environments (hospitals, schools, restaurants, etc.), photocatalysts must display efficient antimicrobial activity under low light intensity. Fu et al. [246], in their instructive study, compared inactivation of *E. coli* and *B. megaterium* under room light and UV light emitted by a 1.0 mW/cm^2^ LED, over Au-capped, 12–18 nm spherical anatase particles, prepared by sol–gel, and deposited on glass slides. The survival ratio as a function of time, presented in Figure 25B, indicates a fast and pronounced killing efficiency of *E. coli* due to the strong oxidizing ability of TiO_2_-Au composites, deposited on glass slides, under low-intensity fluorescent UV light and even room light. Of course, in control experiments without a photocatalyst, inactivation of *E. coli* does not occur.

### 4.5. Visible-Light-Responsive TiO_2_

The requirement for UVA irradiation limits indoor use of TiO_2_ in photocatalytic disinfection. Several strategies to obtain visible-light-responsive TiO_2_ are currently the subject of extensive research, including doping with metal ions and non-metals (C, N, and S), the formation of heterostructures with semiconductors, sensitization with dye molecules, and, most recently, the formation of interfacial charge transfer (ICT) complexes, mainly with colorless benzene derivatives. We will omit from this section the discussion concerning TiO_2_-based composites with noble metal particles, as the mechanism responsible for improved optical properties and their use in pathogen inactivation is provided in the previous section. The review by Girish Kumar and Gomathi Devi [256] offers physical insights into charge transfer events, including charge carrier generation, trapping, detrapping, and transfer across the interface of modified TiO_2_, as well as the proposed theories underlying these processes. Future studies should adopt standardized reporting of all relevant irradiation parameters, such as wavelength range, light intensity, total exposure time, and irradiation dose, to ensure reproducibility and enable reliable comparison of TiO_2_-based photocatalytic antibacterial results.

#### 4.5.1. Dopped TiO_2_

**Figure 26:**
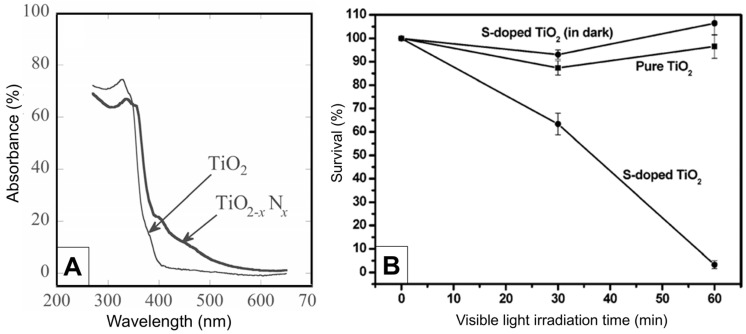
(**A**) Experimental optical absorption spectra of N-doped and pure TiO_2_ films. (**B**) Survival ratio of *M. lylae* vs. visible light irradiation time (>420 nm) for pure and S-doped TiO_2_ (~2 at.-%) powders [259].

Groundbreaking research by Asahi et al. [257] at the beginning of this century emphasized doping with non-metals as a viable approach for creating visible-light-absorbing TiO_2_ that can facilitate the photocatalytic decomposition of organic molecules under visible light excitation. The red-absorption shift in the N-doped TiO_2_ film in comparison to the pristine TiO_2_ film is displayed in Figure 26A. According to XPS measurements, Chen and Burda [258] proposed that visible-light absorption of TiO_2_ doped with non-metals (C, N, and S) is a consequence of extra electronic states above the valence band edge of pure TiO_2_. In general, doped TiO_2_ exhibits an absorption red shift that increases in the order of C > N > S. Also, extra electron density of states explains the lower oxidation potentials of doped TiO_2_.

Several studies conducted a few years after the initial paper by Asahi et al. [257] further explored the possibility of photocatalytically inactivating pathogens under visible light excitation of doped TiO_2_ [259,260,261,262,263,264,265]. Among those studies, the first one by Yu et al. [259] is instructive. Figure 26B displays time-dependent inactivation of Gram-negative *M. lylae* bacteria over S-doped TiO_2_ illuminated with visible light (>420 nm). These authors correlated the considerable bactericidal effect with the generation of hydroxyl radicals induced by the visible light excitation of S-doped TiO_2_, as confirmed by EPR measurements. Of course, inactivation of Gram-negative *M. lylae* bacteria was not observed in control experiments, i.e., contact of bacteria with S-doped TiO_2_ in the dark and upon illumination of pure TiO_2_ by visible light.

Chronologically, the use of doped TiO_2_ with transition metals and rare earth elements for inactivating pathogens began approximately a decade after the use of doped TiO_2_ with non-metals [29,266,267,268,269,270,271,272,273,274,275]. The pronounced photoluminescent properties of transition metals and rare earth elements, hosted by wide bandgap metal oxides, serve as the foundation for many diverse applications of these materials in optoelectronics (such as LEDs and displays), biomedicine (including imaging and localized heating), and non-contact thermometry, so the research focus on environmental remediation, specifically photocatalytic degradation of pollutants and inactivation of pathogens, came later on [29,266,267,268,269,270,271,272,273,274,275].

**Figure 27:**
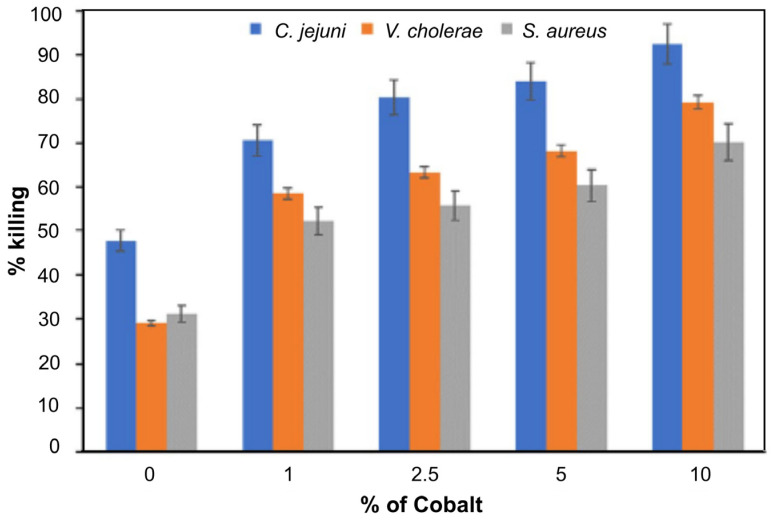
Increase in killing percentage against Gram-positive (*S. aureus*) and Gram-negative (*V. cholerae* and *C. jejuni*) bacteria as a function of dopant concentrations under the same photocatalytic experiments using Co-doped TiO_2_. Adapted from [273].

Besides enhancing the light harvesting ability of TiO_2_ due to the formation of new energy levels near the conduction band, the incorporation of dopants (transition metals and rare earth elements) into the TiO_2_ lattice inhibits grain growth since the substitution of Ti^4+^ with dopant ions frequently results in the appearance of oxygen vacancies and surface defects, and in addition, prevents the recombination of photogenerated charge carriers since dopant ions of less charge than titanium ions in the TiO_2_ lattice act as trap centers. Table 5 summarizes data from references [264,270] concerning the influence of dopant concentration on crystallite size. An increase in dopant concentration induces a decrease in crystallite size, accompanied by a rise in specific surface area and improved charge separation of electron-hole pairs, enhancing the photocatalytic ability of doped TiO_2_, as demonstrated by inactivation of various pathogens using Co-doped TiO_2_ (Figure 27).

Studies concerning the influence of transition metals and rare earth elements on the antimicrobial properties of TiO_2_ have been conducted in parallel, accompanied by attempts to improve photocatalytic inactivation of pathogens using either dual-doped TiO_2_ or heterostructures between doped TiO_2_ and noble metals, primarily silver. Table 6 summarizes the literature data on the antimicrobial activity of the above-mentioned TiO_2_-based photocatalysts operating under visible light excitations [276,277,278,279,280,281,282,283,284,285,286]. Typically, in these studies, the use of nitrogen and inexpensive metals for the preparation of dual-doped TiO_2_ prevails. On the other hand, the noble metal component of heterostructures was silver, which is the least costly noble metal. The Gram-negative bacterium *E. coli* was the most commonly used pathogen for testing the photocatalytic antimicrobial ability of dual-doped TiO_2_ and doped-TiO_2_/Ag heterostructures, frequently accompanied by the Gram-positive bacterium *S. aureus*.

The recent approach for enhancing photocatalytic antimicrobial efficiency relies on the use of up-conversion nanomaterials, which can convert low-energy photons (such as near-infrared light) into high-energy photons (like visible light). Typically, the up-conversion materials consist of a wide-bandgap host doped with lanthanide ions that have an energy level arrangement, which allows multiple absorptions of low-energy photons, followed by emission of a single high-energy photon. The significant application of up-conversion materials is in biomedicine, since “spectral windows” in biology, where light can penetrate biological tissues without notable absorption and scattering, are in the near-infrared region, enabling non-invasive diagnostic processes and therapeutic applications.

In their initial paper, published in 2014, Cates et al. [287] demonstrated the ability of visible-to-ultraviolet C light up-converting yttrium silicate (Y_2_SiO_5_) powders doped with Pr^3+^ and Li^+^ to inactivate *Bacillus subtilis* spores under diffuse fluorescent light. A few years later, Ren et al. [288] reported enhanced photocatalytic disinfection after doping TiO_2_ with Ce and Er. Doping of TiO_2_ with Er provides the possibility to convert photons from the near-infrared to the visible range, while doping with Ce decreases the bandgap energy of TiO_2_, which makes it possible for TiO_2_ to harvest not only UV but also visible light. In addition, both Ce and Er ions suppress the recombination of photogenerated charge carriers due to the incompletely occupied 4f and 5d electron orbitals.

**Figure 28:**
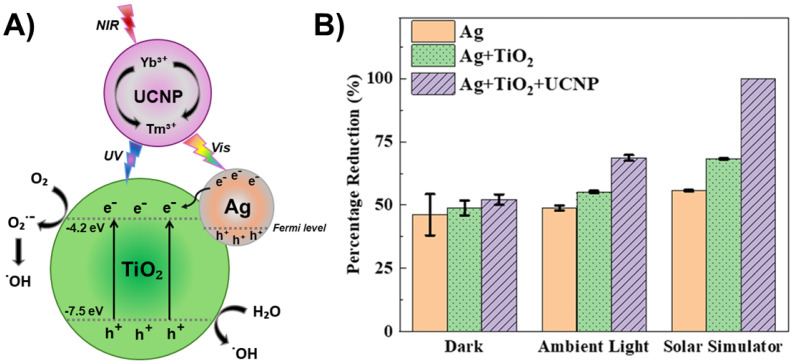
(**A**) Antibacterial mechanism of the ternary nanocomposite consisting of Ag, TiO_2_, and up-converting β-NaYF4@Yb:Tm (UCNP) nanoparticles. (**B**) Comparison of antibacterial performance of Ag nanoparticles, Ag/TiO_2_ nanocomposites, and Ag/TiO_2_/UCNP nanocomposites against *E. coli* in the dark, under ambient light, and a solar simulator equipped with a UV filter (i.e., removal of UV photons) [291].

Recent publications [289,290] provide insight into complex three-component composites consisting of photocatalytic TiO_2_, noble metals (Au or Ag), and up-conversion particles, which serve as a visible light source by converting near-infrared light. For example, Zhang et al. [291] reported superior antimicrobial activity against *E. coli* over a ternary multifunctional composite consisting of TiO_2_, biocidal Ag, and dual-doped sodium yttrium fluoride (NaYF_4_) with ytterbium (Yb) and thulium (Tm) under ambient light and solar simulator irradiation with UV photons filtered, also displaying pronounced photocatalytic ability for oxidation of organic molecules. A schematic presentation of the antibacterial mechanism of this complex architecture is shown in Figure 28A. On the other hand, bar graphs (Figure 28B) indicate synergy between components of the ternary composite, which is manifested by its better antibacterial performance compared with Ag nanoparticles or the TiO_2_-Ag heterostructure in the dark, as well as under ambient illumination or light mimicking the solar spectrum with the UV component filtered out. While the science behind the antibacterial ability of the ternary multifunctional composites is sound, the cost–benefit analysis is an obstacle to their applications.

#### 4.5.2. TiO_2_-Semiconductor Heterostructure

**Figure 29:**
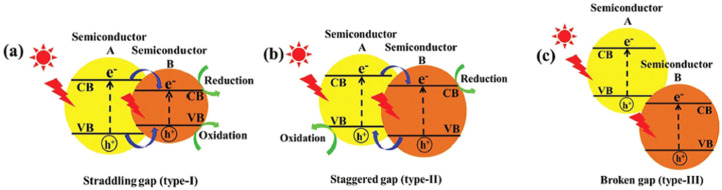
Schematic illustration of electron-hole pairs separation in three light-responsive types of heterojunctions: (**a**) type-I, (**b**) type-II, and (**c**) type-III [291].

Based on the conduction and valence band (CB and VB, respectively) alignments between two semiconductors, there are three types of heterojunctions: those with a straddling gap (type-I), a staggered gap (type-II), and a broken gap (type-III), as shown in Figure 29 [291]. The primary advantage of using heterostructures in photocatalytic processes is the enhancement of charge carrier separation, as discussed in the case of commercial Degussa P25, which comprises anatase and rutile crystal phases (Section 4.2.1). Energy level alignment in a type-I heterojunction leads to the accumulation of photogenerated electrons and holes in the lower bandgap semiconductor (semiconductor B in Figure 29a), whose CB and VB are within the bandgap of the larger bandgap semiconductor (semiconductor A in Figure 29a). In a type-III heterostructure, the migration of charge carriers followed by their separation does not occur because there is no bandgap overlapping of the two semiconductors (Figure 29c). Consequently, type-I and type-III heterojunctions cannot improve the separation of photogenerated electron-hole pairs.

The type-II heterojunction improves the spatial separation of electron-hole pairs because the photogenerated electrons in semiconductor A will migrate to semiconductor B, and the photogenerated holes in semiconductor B will move in the opposite direction, to semiconductor A. This migration process occurs because the CB and VB levels of one semiconductor (A in Figure 29b) are higher than the corresponding levels of the other semiconductor (B in Figure 29b). However, because oxidation reactions occur on the semiconductor with a lower oxidation potential (A in Figure 29b) and reduction reactions occur on the semiconductor with a lower reduction potential (B in Figure 29b), the redox ability of type-II heterojunction photocatalysts is reduced. If TiO_2_ is one component of the heterostructure, the other component should possess pronounced absorption in the visible in addition to the suitable position of CB and VB levels.

Initially, in the second part of the first decade of this century, inactivation of pathogenic bacteria, primarily *E. coli* and *S. aureus*, under visible light excitation (>420 nm) was investigated using heterostructures between TiO_2_ and silver halides, AgBr [292,293,294] and AgI [295]. In the following studies, the choice of TiO_2_ counterparts in heterostructures diversified, including In_2_O_3_ [296] and ZnO [297], although the use of coexisting CuO and Cu_2_O [298,299,300,301,302,303] was more prevalent. Among many studies, the work of Professor Kiwi’s team from Ecole Polytechnique Fédérale de Lausanne [295] provides deep insight into the inactivation mechanism of TiO_2_-based heterostructures operating under visible light excitation.

**Figure 30:**
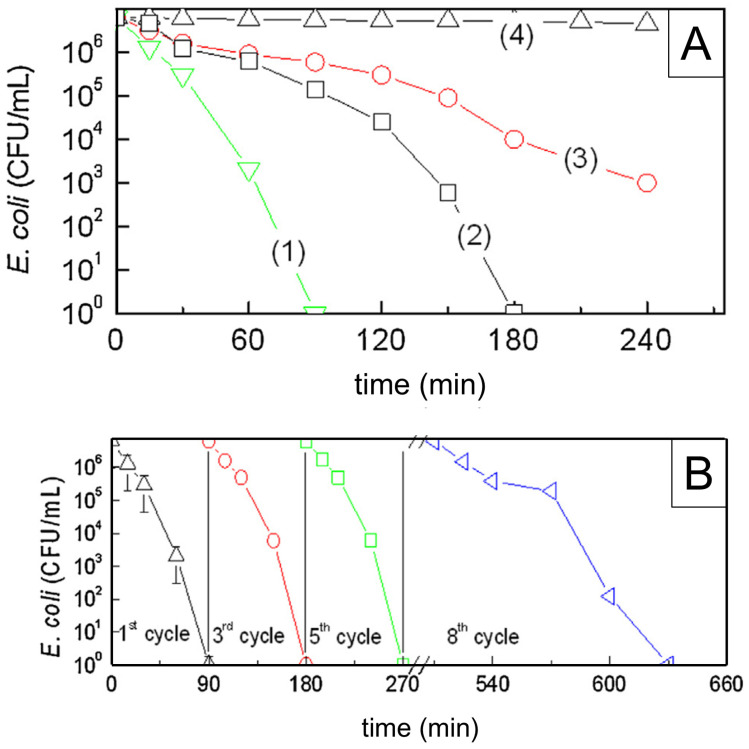
(**A**) Time-kill curves of *E. coli* inactivation over (1) TiO_2_/In_2_O_3_ (green), (2) TiO_2_ (black), and (3) In_2_O_3_ (red), sputtered on polyester, as well as (4) a bare polyester illuminated with a light source frequently used in health facilities (blue). (**B**) *E. coli* inactivation recycling experiments on TiO_2_/In_2_O_3_ deposited on polyester under irradiation by a solar simulator emitting light in the range from 360 to 800 nm [296].

Figure 30A displays the inactivation kinetics of *E. coli* over the TiO_2_/In_2_O_3_ heterostructure and its components, TiO_2_ and In_2_O_3_, deposited onto polyester and illuminated with a light source widely used for lighting health facilities [295]. In_2_O_3_ has a bandgap of 2.7 eV, allowing the TiO_2_/In_2_O_3_ system to absorb visible light. As a result, the photocatalytic inactivation kinetics of *E. coli* are accelerated compared to TiO_2_ and In_2_O_3_ alone. The relative position of energy levels in TiO_2_/In_2_O_3_ corresponds to type-II (staggered gap) alignment, where In_2_O_3_ has higher CB and VB levels of In_2_O_3_ than TiO_2_. This results in efficient charge separation, with holes accumulating in In_2_O_3_ and electrons accumulating in TiO_2_.

The disinfection performance of TiO_2_/In_2_O_3_ deposited on polyester in cycling experiments, under illumination with a light source mimicking solar light (360–800 nm), is shown in Figure 30B. The TiO_2_/In_2_O_3_ loses its ability to inactivate *E. coli* in the 8th cycle. Consequently, TiO_2_/In_2_O_3_ is suitable for textile disinfection, considering the durability of its deposits on polyester and the fact that Ti and In are nontoxic to human health and abundant in nature.

**Figure 31:**
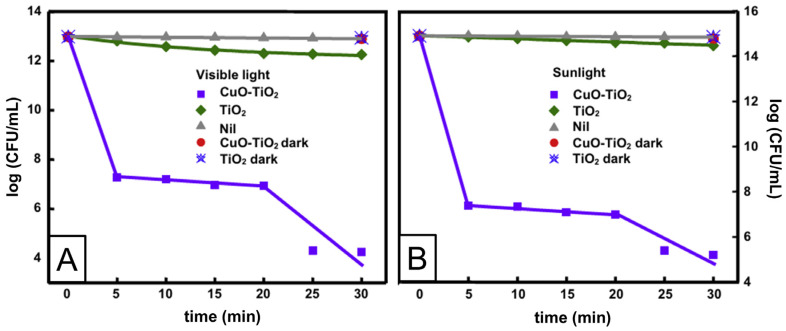
Kinetic curves of *E. coli* inactivation by the TiO_2_/CuO heterostructure under visible light (**A**) and sunlight (**B**); the control experiments are performed either in the dark or under illumination, with or without the presence of pristine TiO_2_ (Degussa P25) [298].

Since the enhancement of TiO_2_ photocatalytic activity by direct doping, i.e., replacement of Ti^4+^ ions in the titania lattice with Cu^2+^ ions, is rather difficult because of a considerable difference in their size, the formation of a heterostructure between TiO_2_ and CuO is an alternative. Figure 31 shows the efficient inactivation of *E. coli* by the TiO_2_/CuO heterostructure under visible light (Figure 31A) and sunlight (Figure 31B). The figure also displays control experiments performed either in the dark or under illumination, with or without the presence of pristine TiO_2_ (Degussa P25) [298]. Since the CB of CuO is less cathodic (E_CB_ = −4.96 eV) than that of TiO_2_ (E_CB_ = −4.21 eV) and the VB of TiO_2_ is more anodic (E_VB_ = −7.41 eV) than that of CuO (E_VB_ = −6.66 eV), the energy alignment corresponds to a type-I heterojunction (straddling gap), where accumulation of both photogenerated electrons and holes takes place on CuO. So, Karunakaran et al. [298] explained the higher photocatalytic bactericidal efficiency of TiO_2_/CuO compared to TiO_2_ doped with non-metals (N and S) by the effective attachment of *E. coli* to TiO_2_/CuO, which leads to efficient flow of ROS from heterostructures to pathogens. Besides being inexpensive, CuO has wide disinfection applications due to its ability to inactivate pathogens in the dark, similar to silver.

Paschoalino et al. [304] suggested a reaction mechanism for ROS formation under the visible light illumination of the CuO. Photogenerated electrons in the conduction band ($e_{CB}^{-}$) can directly reduce oxygen to superoxide radical anions ($O_{2}^{-•})$, as shown previously (Equation (4)), or reduce Cu^2+^ ions from the CuO lattice (${Cu}_{latt}^{2+}$) to Cu^+^ (${Cu}_{latt}^{+}):$

(18) $$e_{CB}^{-}+{Cu}_{latt}^{2+}\to {Cu}_{latt}^{+}$$

followed by the formation of superoxide radicals, again:

(19) $${Cu}_{latt}^{+}+O_{2}\to O_{2}^{-•}$$

Since the equilibrium between H^+^ and $O_{2}^{-•}$ leads to the formation of $HO_{2}^{•}$, the H_2_O_2_ formation takes place by the disproportionation of two protonated superoxide radicals, as previously described in Section 3.1 (Equation (8)), or in the alternate reaction between $HO_{2}^{•}$ and ${Cu}_{latt}^{+}$:

(20) $${Cu}_{latt}^{+}+HO_{2}^{•}+H^{+}\to {Cu}_{latt}^{2+}+H_{2}O_{2}$$

So, visible light excitation of CuO may induce redox cycles of Cu^+^/Cu^2+^, which are analogous to the behavior of Fe^2+^ ions in the Fenton reaction [304].

#### 4.5.3. Surface-Modified TiO_2_ with Dyes and Interfacial Charge Transfer Complexes

Initial studies of water-splitting reactions by dye-sensitized TiO_2_ under visible light excitation were initiated by Professor Grätzel’s team in 1981 [305], followed later by the discovery of the dye-sensitized solar cells, also known as Grätzel solar cells [41]. In contrast, research on pathogen inactivation, which utilized the enhanced absorption properties of dye-sensitized TiO_2_, started about thirty years later [306,307,308,309,310,311]. Nevertheless, these studies represent only a small fraction of the research compared to efforts focused on the decomposition of organic pollutants and the conversion of solar light to chemical or electrical energy.

**Figure 32:**
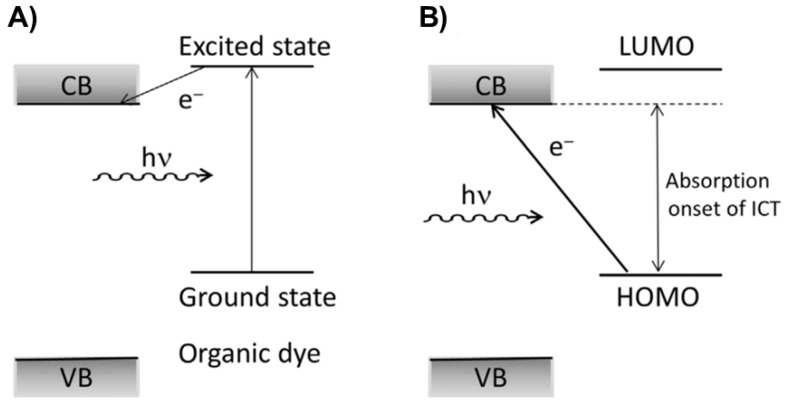
Schematic presentation of electron-transfer in excited (**A**) dye-sensitized TiO_2_ and (**B**) TiO_2_-based interfacial charge transfer complex. (CB—conduction band, VB—valence band).

The photogeneration of charge carriers in dye-sensitized TiO_2_ is a two-step process (Figure 32A). In the first step, the excitation of the dye molecule occurs, followed by the electron transfer from the excited state into the conduction band of TiO_2_ in the second step. Consequently, instantaneous separation of charge carriers into two phases takes place; electrons are delocalized in the conduction band of TiO_2_, while holes are localized on the organic component, the dye molecule. In addition, the optical property of dye-sensitized TiO_2_ is generally an additive function of the optical properties of constituents.

Thin films [306,307,308,311] and nanoparticles [309], including commercial Degussa P25 [310], were the two morphological forms of TiO_2_ mostly used to test the improvement of its antimicrobial performance after anchoring dye molecules to its surface. The dye molecules used for these TiO_2_ modifications were either porphyrins [306,309,311], phthalocyanines [307,310], or azo dyes [308]. In most studies, *E. coli* served as the commonly used pathogen for evaluating antimicrobial activity.

**Figure 33:**
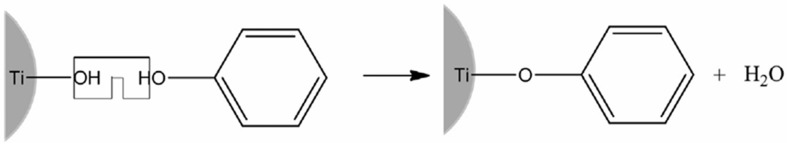
A schematic presentation for the formation of the TiO_2_-based ICT complexes.

Due to the possibility of tailoring materials’ optical properties, particularly TiO_2_, after the initial study by Rajh et al. [148], showing a significant red absorption shift in surface-modified TiO_2_ with vitamin C, the area of the interfacial charge transfer (ICT) complexes has begun to develop rapidly [149,312,313]. The formation of TiO_2_-based ICT complexes is facilitated by a polycondensation reaction between the hydroxyl groups originating from the surface of the TiO_2_ and the colorless aromatic molecules, as sketched in Figure 33.

The creation of ICT complexes as a method to bring the absorption of TiO_2_ to a more useful spectral range, visible or near-infrared, is in many ways beneficial in addition to being a straightforward synthetic process. First, there is a strong covalent linkage between the inorganic and organic components of the ICT complex (Ti–O–C). Second, as noted by Fujisawa et al. [313], the promotion of electrons from the ligand’s ground state to the TiO_2_ conduction band is a one-step process (Figure 32B) without energy loss, opposite to the excitation process in dye-sensitized TiO_2_ (Figure 32A). Third, by selecting the right phenyl-ring-substituted groups, as demonstrated in a groundbreaking work by Higashimoto et al. [292], the optical characteristics of TiO_2_-based ICT complexes can be optimized, avoiding a trial-and-error method. To put it simply, ligands with free electron-donating groups will cause the decrease in the bandgap energy in comparison to ligands without substituent groups, whereas the presence of electron-withdrawing groups causes the energy gap to widen. Furthermore, by modifying the surface charge of inorganic–organic hybrids, free functional groups can enhance electrostatic interactions with cell walls and, as a result, increase the effectiveness of pathogen inactivation. Lastly, the creation of higher hierarchical structures is made possible by the appropriate functionalization of TiO_2_. For instance, a straightforward technique for the in situ reduction of silver ions to metallic silver particles connected to a metal oxide over a ligand [11,314] is provided by the addition of an amino group with a strong reducing ability [315].

While the application of the TiO_2_-based ICT complexes in photocatalytic degradation of organic pollutants and water-splitting reaction is extensively studied and recently reviewed [316], despite proven nontoxicity in in vivo experiments [317,318], there are just a few attempts to take advantage of visible-light-responsive TiO_2_-based complexes to inactivate pathogens [319,320,321], similar to the use of dye-sensitized TiO_2_ in wastewater treatment.

**Figure 34:**
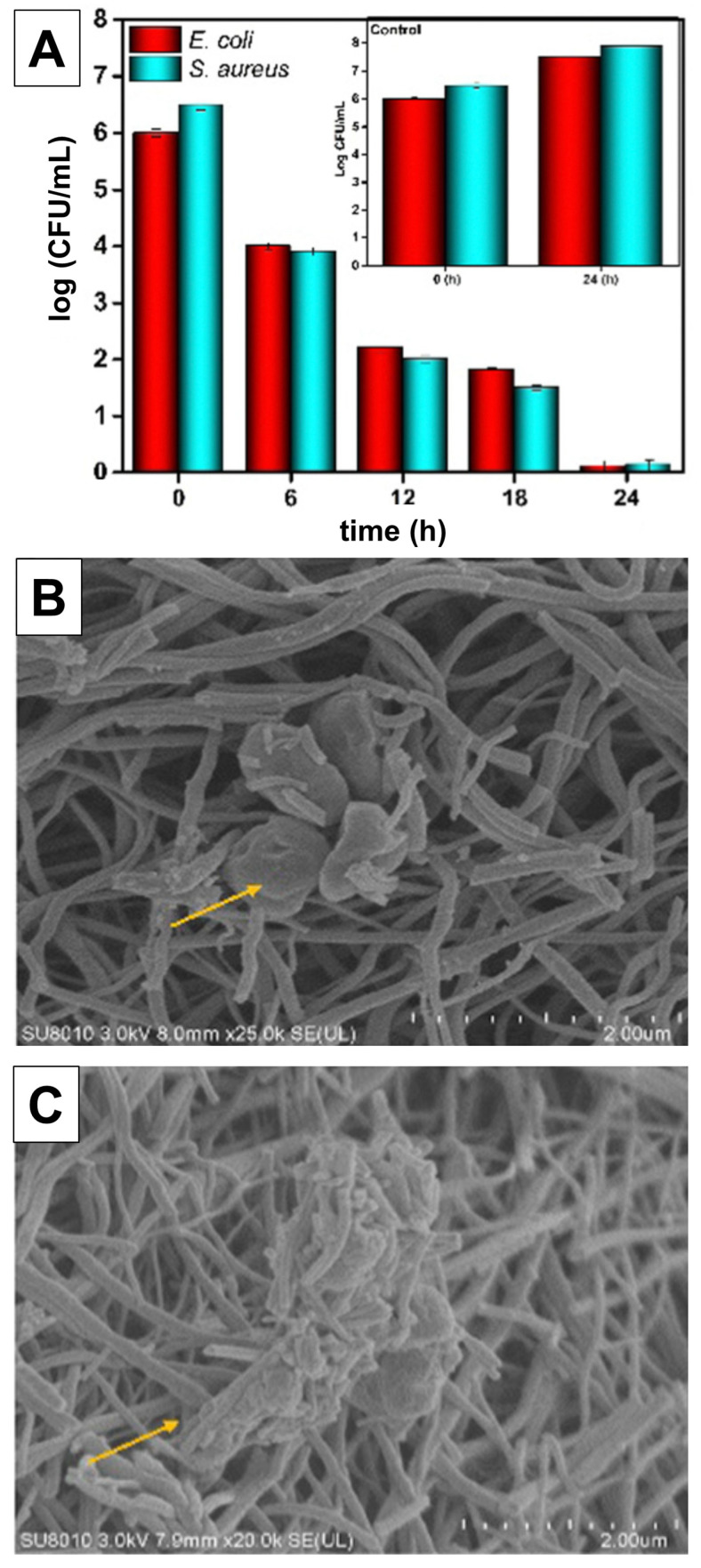
(**A**) Time-dependent antibacterial activity of TiO_2_ nanofibers (NFs) functionalized with rhodizonic acid (RhA) against *E. coli* and *S. aureus* under visible light excitation; control experiments, in the dark, are presented as an inset. (**B**,**C**) SEM images of *E. coli* and *S. aureus*, respectively, after 24 h of contact with functionalized TiO_2_ NFs with RhA under visible light excitation. Scale bar (shown in panels B and C) is 2 μm; bacteria are marked with arrows.

The first proof that TiO_2_-based ICT complexes can inactivate pathogens (*E. coli* and *S. aureus*) under exclusive visible light excitation was presented in the paper of Shahriari-Khalaji et al. [321]. The inactivation kinetics of *S. aureus* and *E. coli* over TiO_2_ nanofibers (NFs) functionalized with rhodizonic acid (RhA) are displayed in Figure 34A. The NFs absorb in the visible spectral range (E_g_ = 2.1 eV) and are activated by a light source that emits only in the visible spectrum. Figure 34 shows the morphologies of *S. aureus* and *E. coli* following a 24 h photocatalytic inactivation process using surface-modified TiO_2_ NFs with RhA (B and C, respectively). Both *S. aureus* and *E. coli* cell walls were broken, which allowed the internal contents of the bacterium to seep out. As a result, the morphologies of *S. aureus* and *E. coli* differ from the usual spherical and rod-like forms, respectively, that were maintained in dark control trials. Therefore, the results derived from counting bacterial colonies are consistent with microscopic, visual observations.

We highlighted the utilization of high-biological-value plant chemicals, such as flavonoids, as an economical and environmentally beneficial method of synthesizing TiO_2_ in Section 4.2.3. Nevertheless, there are currently only a few studies on the bactericidal action of TiO_2_-based ICT complexes with bioactive substances with multiple substituent hydroxyl groups on phenyl rings [320,321]. The antibacterial activity of the ICT combination between commercial TiO_2_ powder and dihydroquercetin (DHQ), a common component of plant extracts, against *E. coli* under visible-light excitation is demonstrated in a recent paper by Nikšić et al. [320]. The coordinated DHQ on the TiO_2_ surface has a protective effect against oxidative stress in addition to improved antimicrobial activity, according to other biological assessments such as oxidative stress, genotoxicity/antigenotoxicity, and cell viability in various cell lines. Because of its improved antioxidant qualities, the TiO_2_-based ICT complex with DHQ may be used as a safe and non-toxic biocide agent.

## 5. Conclusions and Perspectives

Pathogens in the environment, especially those resistant to conventional antibiotics, pose a significant threat to human health, raise healthcare costs, and become an economic burden on patients and society as their numbers rapidly increase due to antibiotic overuse. Although various chemicals used in disinfection practices can inactivate pathogens, their application often replaces one environmental problem with another due to their toxicity. The growing demand for eco-conscious strategies led to the development of various TiO_2_-based photocatalysts, offering a promising and environmentally friendly strategy to inactivate pathogens. Due to favorable physicochemical properties (stability, durability, and corrosion resistance), biocompatibility, reusability, and low cost, TiO_2_ is the most used photocatalyst for bacterial inactivation. An additional advantage of TiO_2_-based photocatalysts lies in the fact that along with pathogen inactivation, the degradation of organic pollutants can take place since the same photogenerated reactive species participate in both processes. Besides using commercial TiO_2_ (Degussa P25), numerous methods have been employed to prepare a variety of TiO_2_ modifications suitable for applications of interest. These methods include sol–gel and green syntheses using plant extracts, thin film formation, nanoparticle immobilization onto polymers, heterostructures with plasmonic noble metals and semiconductors, doping with light and heavy elements, and surface modification with dyes and interfacial charge transfer complexes. The main intention of the preparation of complex TiO_2_-based architectures is to increase the efficiency of photocatalytic pathogen inactivation by overcoming two main drawbacks: the low harvesting ability of light and the inefficient charge separation caused by the recombination of photogenerated electron–hole pairs in TiO_2_ on a picosecond time scale.

Over the past few decades, extensive studies on photocatalytic pathogen inactivation over various TiO_2_-based photocatalysts have been conducted. However, from both a fundamental and practical perspective, some questions remain without proper explanation. The primary challenge at a fundamental level is to understand the mechanism of toxic action, a task that requires researchers with biomedical profiles in this multidisciplinary field. On the other hand, from the perspective of material science, the main limitation of using any catalyst is its stability. For example, research on novel TiO_2_-based photocatalysts operating under visible light excitation, which takes advantage of either the up-conversion effect or the formation of ICT complexes, is still in its embryonic stage. So, neither their efficiency in pathogen inactivation, although high, is optimized yet, nor is their stability under long-time working conditions.

Besides the stability and reusability of TiO_2_-based photocatalysts, the complexity of modification procedures directly influences their cost, and the need for consistent performance in real-world environments remains a practical application challenge. Therefore, further studies are necessary to develop inexpensive TiO_2_-based antimicrobial agents using a straightforward methodology that are efficient due to their low recombination rate of photogenerated charge carriers and the ability to harness visible light. In addition, the environmental impact and safety of any newly invented catalyst are essential factors to consider, i.e., to ensure that they are not hazardous.

Despite their promising antibacterial properties, TiO_2_ nanoparticles also face several inherent limitations. Their photocatalytic activity is predominantly restricted to UV irradiation, which represents only a small fraction of the solar spectrum. In addition, nanoparticle aggregation, potential cytotoxicity, and environmental safety concerns, as well as the lack of standardized testing protocols and limited long-term stability of coatings, pose significant challenges. These factors underscore the need for further optimization of TiO_2_ systems, as well as the development of strategies to strike a balance between high antibacterial efficacy and safety, cost-effectiveness, and practical applicability.

In light of the preceding overview, after the recognition of the ability of TiO_2_ and other wide bandgap oxides to inactivate pathogens in the mid-eighties of the last century, changes in the research focus, as well as their timeline, are easy to follow. The fundamental research, initially conducted, evolved over several years into attempts to apply inexpensive commercial TiO_2_ powders as an active component, dispersed either in water or various solids, for pathogen inactivation on a large scale. The most recent studies are again on a fundamental level, intending to enhance antibacterial efficiency by overcoming the low light-harvesting ability of pristine TiO_2_ and suppressing the recombination of photogenerated charge carriers. Considering, on one side, countless ways to create visible-light-responsive TiO_2_ photocatalysts, either by doping or coordinating ligands to the TiO_2_ surface, and, on the other side, only a few recent preliminary papers showing high antibacterial performance of prepared hybrid materials, this line of research is worthy of further investigation. Of course, the trade-off between efficiency and cost will determine whether or not newly developed TiO_2_-based photocatalysts are suitable for commercial use.

## Figures and Tables

**Table 1:** Antimicrobial activity of commercial TiO_2_ (Degussa P25) suspensions excited by UV light against various microorganisms.

Microorganism	Reference
*Escherichia coli*	[83,84,85,86,87,88,89,90,91,92,93,94,95,96,97,98,99,100,101,102,103,104,105,106]
*Enterococcus faecalis*	[88]
*Enterobacter cloacae*	[90]
*Pseudomonas aeruginosa*	[90,95,98,99]
*Salmonella typhimurium*	[90,106,107]
*Staphylococcus aureus*	[95,105,106]
*Salmonella enteriditis*	[98]
*Bacillus subtilis*	[99,104]
*Pseudomonas fluorescens*	[104]
*Serratia marcescens*	[105]
*Coliforms*	[108,109]
*Streptoccocus faecalis*	[109]
*Erwinia carotovora*	[110]
*Pseudomonas syringae*	[110]
*Streptococcus sobrinus*	[111]

**Table 2:** Overview of the literature data on green synthesized TiO_2_ using plant extracts employed against pathogens.

Precursors		TiO_2_ Properties		Tested Pathogen	Reference
Plant Extract	Source of Ti	Crystal Phase	Morphology		
*Psidium guajava*	TiO(OH)_2_	A+R	spherical, 32.5 nm	*A. hydrophila*, *P. mirabilis*, *E. coli*, *S. aureus*, *P. aeruginosa*	[150]
*Morinda citrifolia*	TiCl_4_	R	spherical, 10–20 nm	*S. aureus*, *E. coli*, *B. subtilis*, *P. aeruginosa*	[136]
*Trigonella foenum-graecum*	TiO(SO_4_)	A	spherical, 20–90 nm	*E. faecalis*, *S. aureus*, *S. faecalis*, *B. subtilis*, *Y. enterocolitica*, *P. vulgaris*, *E. coli*, *P. aeruginosa*, *K. pneumonia*	[142]
*Glycyrrhiza glabra*	TiO(SO_4_)	A	spherical, 60–140 nm	*S. aureus*, *K. pneumonia*.	[143]
*Punica* *granatum*	TTIP	A	various shapes, 1–5 μm	*P. aeruginosa*, *E. coli*, *S. aureus*	[151]
*Aloe barbadensis*	TiCl_4_	A+B+R	spherical, ~20 nm	*P. aeruginosa*	[152]
*Lupin bean*	TTIP	A	spherical (9.2 nm) and nanorods	*Enterococcus*, *E. coli*	[154]
*Mentha arvensis*	TTIP	A	spherical, 20–70 nm	*P. vulgaris*, *S. aureus*, *E. coli*, *A. cuboid*	[155]
*Acorus calamus*	TTIP	A	Spherical, 11–30 nm	*E. coli*, *P. aeruginosa*, *B. subtilis*, *S. aureus*	[156]
*Morus alba*	TTIP	A	spherical, 24 nm	*E. coli*, *S. aureus*	[157]
*Luffa acutangula*	TiO(SO_4_)	R	hexagonal, 10–49 nm	*B. subtilis*, *E. coli*, *E. faecalis*, *K. pneumoniae*, *S. aureus*, *P. aeruginosa*	[158]
*Nervila aragona*, *Ceaspina pulcherrima*, *Manihot esculante*	TTIP	A	spherical, 15–28 nm	*E. coli*, *S. aureus*, *P. aeruginosa*	[147]
*Spinacia oleracea*	TiO(SO_4_)	A	spherical, 38 nm.	*E. coli*, *S. aureus*	[159]
*Fagonia cretica*	TiO(OH)_2_	R	spherical, 20–80 nm	*K. pneumoniae*, *S. aureus*, *P. aeruginosa*, *E. coli*	[141]

**Table 3:** Relative intensity of deconvoluted high-resolution C1 photoelectron peaks for the untreated polyester (U-PES), and oxygen and argon plasma-treated polyesters (O_2_-PES, and Ar-PES) fibers [190].

Sample	Atomic Ratio (%)			
	C–C, C–H	C–O	C=O	O–C=O
U-PES	77.4	13.1	0.0	9.5
O_2_-PES	63.6	21.0	8.3	7.1
Ar-PES	70.4	14.2	10.3	5.1

**Table 4:** Inactivation of *E. coli* over 1 mg/mL TiO_2_ or TiO_2_-Ag composite as a function of illumination time using a low-pressure mercury lamp as a UV light source [218].

Time (min)	Viable Cell Concentration (CFU/mL)	
	TiO_2_	TiO_2_-Ag
0	1 × 10^7^	NVC
10	1 × 10^6^	NVC
15	0.5 × 10^6^	NVC
20	NVC	NVC
30	NVC	NVC
40	NVC	NVC

NVC—no viable cell.

**Table 5:** Crystallite size of doped TiO_2_ as a function of dopant concentration [267,273].

Co-Doped TiO_2_		Fe-Doped TiO_2_	
Dopant Concentration (mol.-%)	Crystallite Size (nm)	Dopant Concentration (vol.-%)	Crystallite Size (nm)
0.0	14.1	0.0	26.1
1.0	12.2	3.0	24.8
2.5	12.0	4.0	22.9
5.0	8,0	5.0	20.2
10.0	7.1	6.0	19.5

**Table 6:** Overview of the literature data on antimicrobial activity of dual-doped TiO_2_ or heterostructures between doped TiO_2_ and noble metals operating under visible light excitations.

Dopants		Heterostructure with Noble Metal	Pathogen	Reference
Non-Metal	Metal Ion			
N		Ag	*E. coli*, *B. subtilis*	[276]
C, S		Ag	*E. coli*, *B. subtilis*	[277]
N	Cu		*E. coli*, *E. faecalis*	[278]
N		Ag	*E. coli*	[279]
N, F			*E. coli*, *S. aureus*,	[280]
N	Ni		*E. coli*, *S. aureus*	[281]
S	Mn		*B. coagulans*, *K. pneumoniae*	[282]
N	Cu		*E. coli*, *S. aureus*	[283]
N	Co		*E. coli*, *S. aureus*, *L. pneumophila*	[284]
S	Co		*E. coli*, *S. aureus*	[285]
	Fe	Ag	*E. coli*, *S. aureus*	[286]

## Data Availability

No new data were created or analyzed in this study. Data sharing is not applicable to this article.
